# Human visual search follows a suboptimal Bayesian strategy revealed by a spatiotemporal computational model and experiment

**DOI:** 10.1038/s42003-020-01485-0

**Published:** 2021-01-04

**Authors:** Yunhui Zhou, Yuguo Yu

**Affiliations:** 1 School of Life Sciences, Fudan University, 200433 Shanghai, China; 2 State Key Laboratory of Medical Neurobiology and MOE Frontiers Center for Brain Science, Fudan University, 200433 Shanghai, China; 3 Human Phenome Institute, Fudan University, 200433 Shanghai, China; 4 Research Institute of Intelligent Complex Systems and Institutes of Brain Science, Fudan University, 200433 Shanghai, China; 5 Institute of Science and Technology for Brain-Inspired Intelligence, Fudan University, 200433 Shanghai, China

**Keywords:** Object vision, Human behaviour, Computational neuroscience

## Abstract

There is conflicting evidence regarding whether humans can make spatially optimal eye movements during visual search. Some studies have shown that humans can optimally integrate information across fixations and determine the next fixation location, however, these models have generally ignored the control of fixation duration and memory limitation, and the model results do not agree well with the details of human eye movement metrics. Here, we measured the temporal course of the human visibility map and performed a visual search experiment. We further built a continuous-time eye movement model that considers saccadic inaccuracy, saccadic bias, and memory constraints. We show that this model agrees better with the spatial and temporal properties of human eye movements and predict that humans have a memory capacity of around eight previous fixations. The model results reveal that humans employ a suboptimal eye movement strategy to find a target, which may minimize costs while still achieving sufficiently high search performance.

## Abstract (web summary)

Yunhui Zhou and Yuguo Yu propose a continuous-time eye movement model capable of predicting both eye fixation location and duration. Their model accounts for saccadic inaccuracy/bias and memory constraints and, applied to real data, shows that humans may use an eye movement strategy that balances task performance and costs when searching for a target.

## Introduction

For animals with foveated retinas, efficient visual search is important for survival and requires planning of eye movements both in space and time. Previous studies on human eye movements have resulted in conflicting evidence as to whether these eye movements are spatially optimal^1–6^. The Bayesian ideal searcher^3^ and the closely related entropy-limit-minimization (ELM) model^1^ are two important optimal eye movement models of multi-saccade visual search tasks. While some studies have shown that human search performance and eye movement statistics are consistent with these optimal models^1–3^, humans seem to make fewer long saccades (rapid eye movements between fixation points) compared to the optimal models^1^. Moreover, other studies have indicated that humans may rely more on suboptimal strategies rather than calculating and planning optimal eye movement for a specific task^4–7^. There is also evidence for statistical dependencies between successive eye movements during visual search^8^, but detailed comparisons to the optimal models are still missing.

The Bayesian ideal searcher and the ELM model are optimal in the sense of fully using the visibility map^1,3^, but they do not consider other costs required to perform the task. For humans, the assumption of unlimited memory capacity in these optimal models is unrealistic, and making eye movements also has costs. For example, longer saccades are less accurate and increase the chances of making a secondary saccade^9^, take longer to finish^10^, and disrupt vision during saccade more severely^11^. This may explain why humans make fewer long saccades than the optimal models^1^. With these constraints, it is unlikely that humans strictly follow the optimal search rule. We therefore reconsidered the question of how optimally humans search and ask if a visual search model with more biological limitations could provide more accurate predictions.

Moreover, the temporal control of eye movements has received surprisingly little attention in visual search models. For example, the Target Acquisition Model does not explain fixation duration^12^; the Bayesian ideal searcher and ELM model assume each fixation last for 250 ms^1,3^; and the Guided Search model assumes fixation duration is 200–250 ms^13^; whereas the fixation duration observed in experiments usually ranges from 100 to 700 ms^14^. We found only one model of visual search that considered mean fixation duration, but it could not simulate the whole distribution of fixation duration^15^. The lack of fixation durations in the above models may reflect the view that visual search is more about deciding fixation locations than fixation duration. However, since it has been shown that there is interdependency between the spatial and temporal control of eye movements^8,16,17^, a complete model of visual search should explain both aspects instead of focusing on a single one.

Eye movements are typically viewed as sequence of decisions, and fixation duration can be viewed as reaction time of a saccade decision. Indeed, the distribution of fixation duration shows a similar right-skewed shape to the reaction time distribution of other decision-making tasks^18–20^. Therefore, some models of reading and scene viewing^21–24^ capture the fixation duration distribution by using the drift-diffusion model of decision-making, which is widely used in explaining the reaction time distribution^25^. The central idea is to accumulate information stochastically within a fixation and trigger a saccade whenever the accumulation reaches a threshold, and such accumulation process can be observed in experiment^26^. Although this approach is successful, the accumulation process in these models are usually simplified or artificial as they do not quantitatively measure the dynamics of information accumulation within a fixation. Since previous studies have shown that visual search task difficulty affects fixation duration^27,28^, we believe that the information accumulation process should be explicitly measured according to the visual search stimulus used in the task, and the data can be incorporated to previous optimal models^1,3^ based on signal detection theory.

In this study, we experimentally measured the accumulation of visual information within a single fixation and formulated a drift-diffusion process that decides saccade timing. Combined with the ELM model, we proposed a continuous-time eye movement model that determines both fixation location and duration. We find that human eye movement statistics are inconsistent with the optimal models. Instead, adding constraints on saccade accuracy, saccade amplitude and memory capacity improves the model’s predictions of human eye movement statistics while still achieving a high search performance.

## Results

### Selecting target contrast

**Fig. 1:**
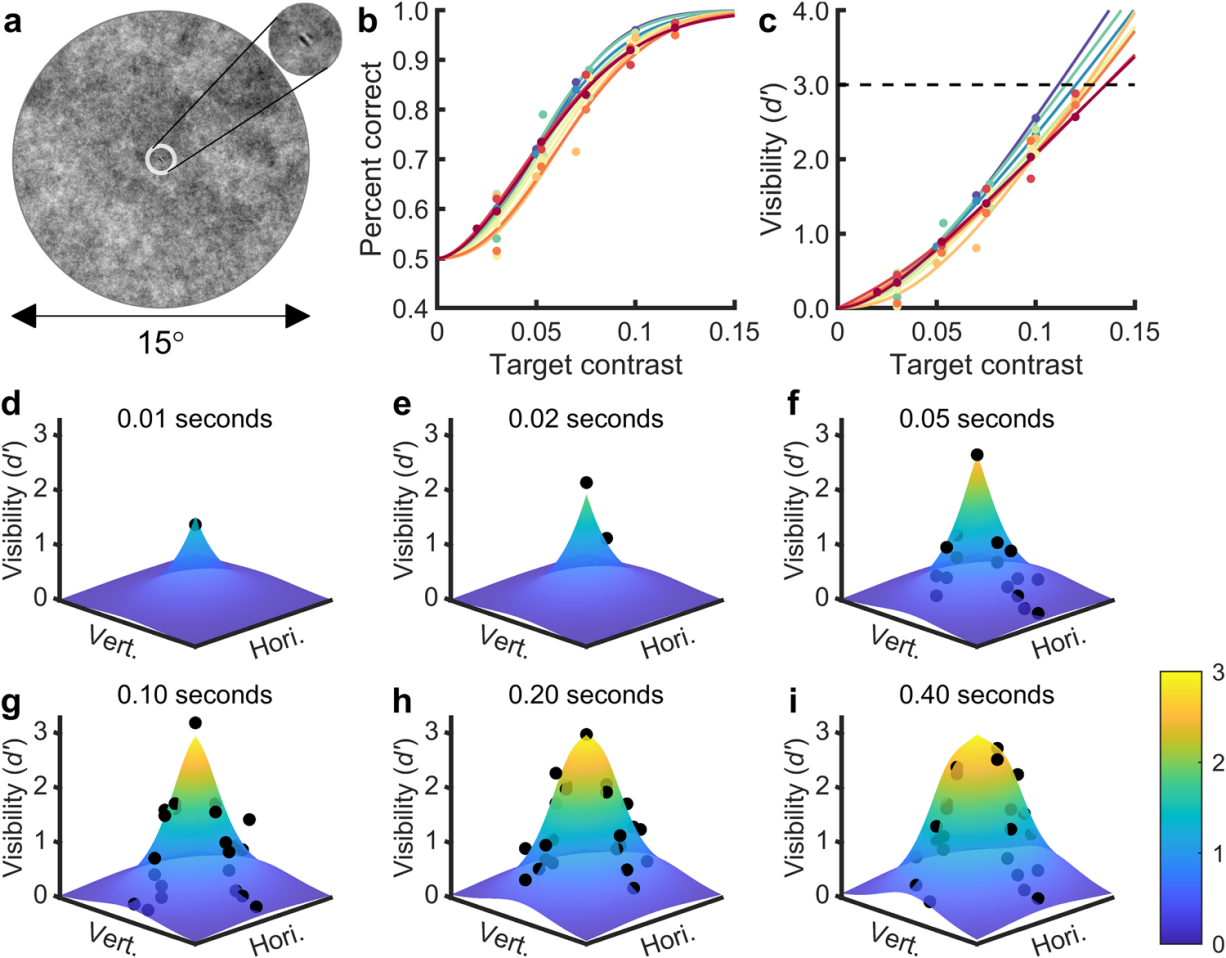
Stimulus image and results of the detection task. **a** The stimulus image was a 1/*f*^*2*^ noise image (15° diameter, root-mean-squared (RMS) contrast 0.2) with an embedded target. The target was a 6 cycle/degree Gabor grating with a diameter of 0.3°, oriented 45° counterclockwise from the vertical and windowed by a symmetrical raised cosine (half-height width of one cycle). The area outside the image was set to the mean gray value of the image (0.5, not shown here). **b**, **c** Detection rate (**b**) and foveal target visibility (**c**) as a function of the target RMS contrast. Dots are raw data (one color per subject). In **b**, the curves are the Weibull function fit to the dots. In **c**, the curves are calculated from Weibull functions fit to hit rates and the correct rejection rate of the raw data (Eq. (1)). The intersection between the dashed line (*d*^′^ = 3.0) and the curves is the target RMS contrast chosen for each subject. **d**–**i** Target visibility map at different stimulus exposure times in the detection task. The dots are raw data merged from four subjects. The surface is the fitted visibility map function (Eqs. (10)–(12)). Not all locations were tested in all exposure times so the number of dots may differ in each subplot. Hori/Vert: horizontal/vertical dimension of the search field. Fixation location was at the center of search field.

Ten student volunteers (six male, four female) participated in the experiment. The stimulus image was a Gabor target (6 cycle/degree, 0.3° in diameter) embedded in a circular naturalistic background noise image (15° in diameter, root-mean-squared (RMS) contrast 0.2) (Fig. 1a). To normalize visual search difficulty across subjects, we first measured the relationship between foveal target visibility (*d*’ in signal detection theory) and target RMS contrast. We presented two noisy images for 250 ms each, and a randomly chosen image had the target at the image center. The subjects needed to identify the image that contained the target (Supplementary Fig. 1a). The target contrast varied in each trial. The subjects’ correct response rate could be well fitted by a Weibull function given the target RMS contrast (Fig. 1b). We then fit the relationships between the hit and correct rejection rates and the target RMS contrast with a Weibull function, and mathematically described the relationship between the target RMS contrast and *d’* (Eq. (2), Fig. 1c). For each participant, we selected the target RMS contrast that yielded *d*′ = 3.0 (Fig. 1c). The selected contrast ranged from 0.111 to 0.135, with a mean value of 0.125.

### Temporal course of target visibility

We then measured how the target visibility changed over time within one fixation across the visual field. We again presented two noisy images to the subjects, but this time, the target contrast was fixed to the selected value for each subject, and the stimulus presentation time and target location varied across trials. Before the start of each trial, the target location was cued, and the subjects needed to keep fixating on the image center throughout the trial. The task was to identify the image that contained the target at the cued location (Supplementary Fig. 1b). Four of the ten subjects performed this experiment, and the trials were pooled together to calculate the visibility at each combination of the visual field locations and stimulus presentation times.

We next built an evidence accumulation model that could generate this measured visibility map. The model assumed that in each short time interval, the visual system received a small normally distributed random sample of visual information at each location (Eq. (3)), and these samples were integrated over time in a leaky manner (Eq. (6)). This produced a drift-diffusion process, and the distribution at each time point could be analytically described (Eqs. (7)–(9)). According to signal detection theory, visibility (*d*′) is the separation between the noise and target + noise distributions in units of their common standard deviation (SD)^29^, therefore the temporal course of visibility could be described (Eqs. (10)–(12)).

In all subjects, the target visibility first increased as a function of stimulus presentation time and then reached stable peak values (Supplementary Fig. 2). The steady-state foveal visibility was close to the predefined value of 3.0. The steady-state and rising speed of visibility was higher in central than in peripheral visual field (Supplementary Fig. 3). There was some individual variability on the spatial span of visibility map (the ability to detect a distant peripheral target), but the trend that central vision could detect the target faster than peripheral vision was common. The temporal course of visibility across the visual field could be described by the evidence accumulation model after fitting to the pooled data from all subjects (Fig. 1d–i).

### Human eye movement strategy in visual search

We next performed a visual search experiment using the same stimulus images (Supplementary Fig. 1c) to compare the eye movement data to visual search models. All ten subjects finished this task, and they were split into training set (four subjects that measured the temporal course of visibility) and testing set (the rest six subjects). The quality of the eye-tracking data of all the subjects was quite good. On average, the SD of the samples of 99.77% of the fixations was below 0.4°, and the RMS of the inter-sample angular distance of 99.50% of the fixations was below 0.03° (Supplementary Fig. 4).

**Fig. 2:**
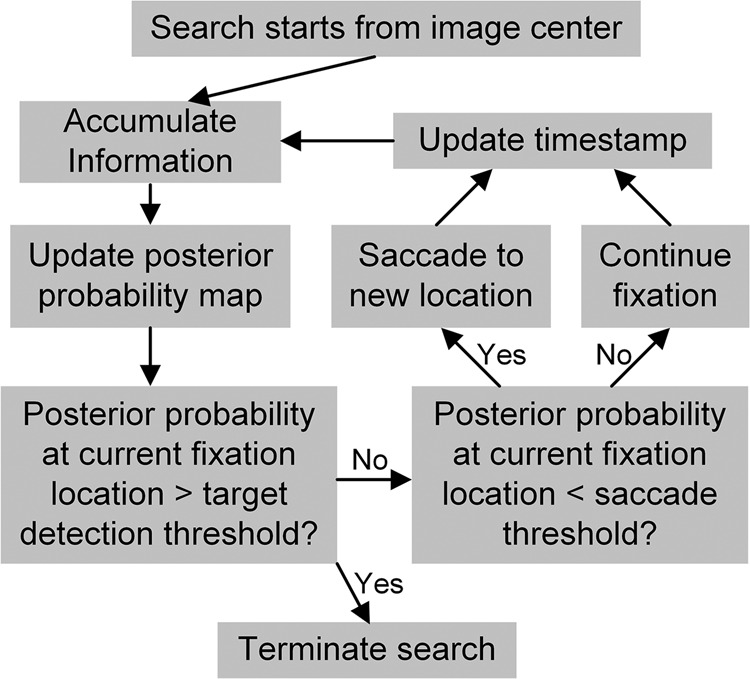
Schematic diagram of the continuous-time entropy-limit minimization (CTELM) and constrained-CTELM (CCTELM) models. The model starts each trial by fixating at the image center. Then, at each time step, it accumulates the visual information sampled since the start of the current fixation, and calculates the posterior probability map of target location across the search field by Bayesian theory. If the posterior probability of target being at current fixation location is larger than a target detection threshold, the model will set the target location as the current fixation location and terminate search. Otherwise, if the posterior probability at current fixation location is lower than the saccade threshold, it will saccade to a new location. If neither of the thresholds are met, the model will continue the current fixation. The whole process iterates until the model finds the target.

We used the ELM model of visual search as the baseline model and it served as a replication of previous study^1^. To model eye movements control both in space and time, we combined the evidence accumulation model and the ELM model into a continuous-time ELM (CTELM) model. Since several previous studies have suggested that the control of fixation duration depends more on foveal than peripheral visual analysis^30,31^, the CTELM model terminates a fixation when the posterior probability of target being at current fixation location drops below a collapsing threshold (Fig. 2). The next fixation location was chosen within 400 predefined locations (Supplementary Fig. 5a) by maximizing the expected information gain of the next fixation (Eq. (18)).

We also tested whether adding constraints to the CTELM model (called the CCTELM model) could improve model’s prediction on experiment data. Three constraints were considered: preference for short saccades, inaccuracy of saccade landing position, and memory limitation. We applied a penalty function (Eq. (26)) when calculating the expected information gain of the next fixation to suppress the probability of choosing a distant saccade target. The relationship between the bias and variance of saccade landing position and saccade target eccentricity was obtained from a previous study^19^ (Supplementary Fig. 6), and the model could fixate at any location instead of the predefined 400 locations. We also considered a simple form of memory so that the model could only integrate a fixed number of fixations when calculating the posterior probability of target location (Eq. (24)). Visual information prior to what the memory could hold was discarded. The model’s goodness-of-fit was quantified by calculating Bhattacharyya coefficient^32^ (*B*_c_, Eq. (32)) of the distributions of eye movement metrics. The parameters in the models were fit to the training set and tested against the testing set. We found that human eye movements predicted that the memory capacity was about eight fixations (including the current fixation). In the following sections we will show how we estimated the capacity.

**Fig. 3:**
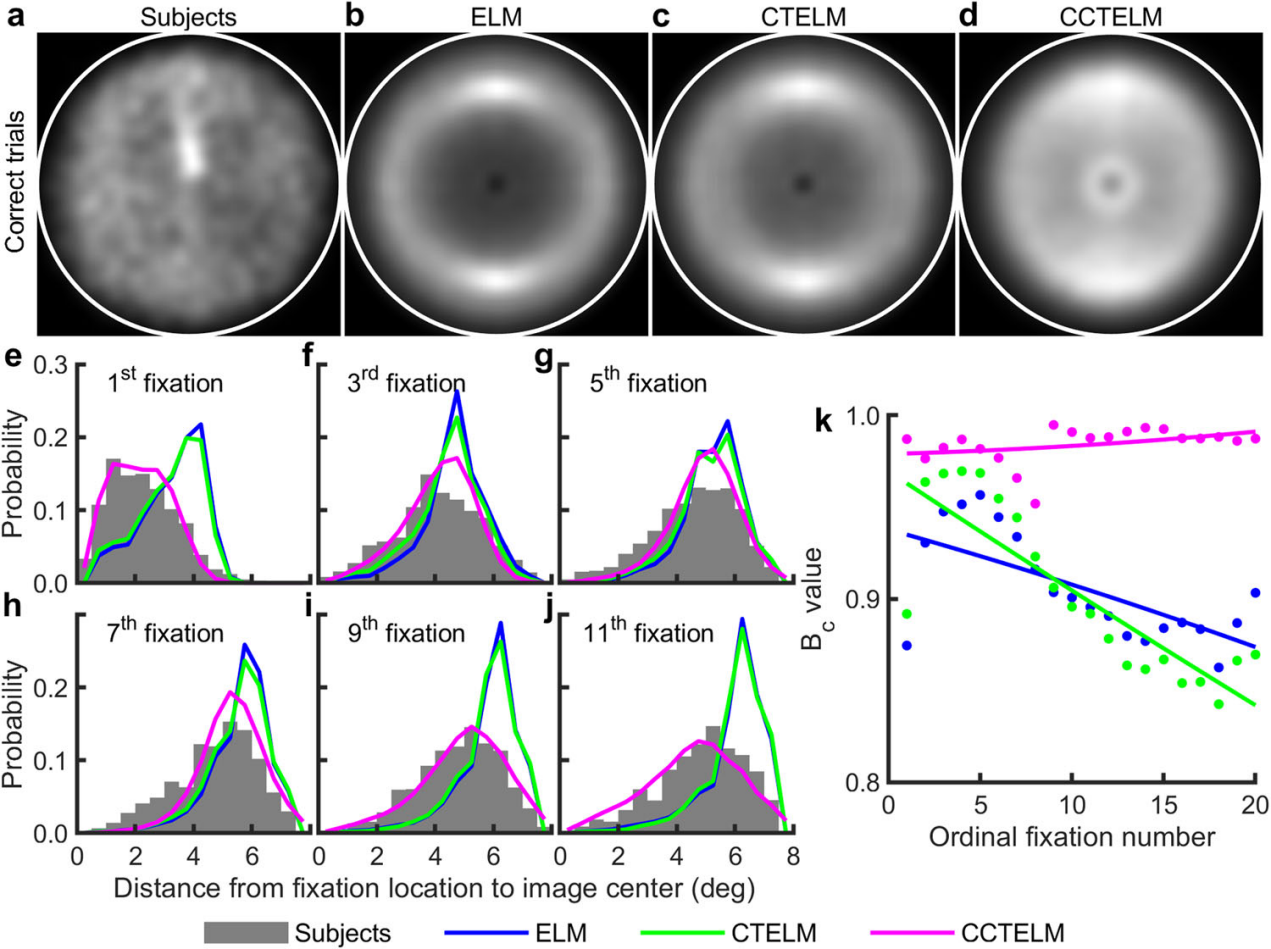
Fixation location distribution of subjects and models in correct trials. **a**–**d** Distribution of fixation location in the search field (inside the white circle), lighter means higher density. The densities were obtained by smoothing the scatterplot of the fixation locations by a Gaussian window with a SD = 0.35° and then normalizing the maximum value in each subplot to one. **e**–**j** Fixation distance distribution to image center within the initial 11 fixations after the first saccade. **k** Bhattacharyya coefficient (*B*_c_) between models’ and subjects’ distributions of fixation distance to image center of the initial 20 fixations after the first saccade. Dots represent raw data; curves represent quadratic functions fitted to the dots.

We first examined how the three models predict subjects’ spatial control of eye movements. In previous studies of similar experiment, humans produced a doughnut-shaped fixation location distribution peaked at about 5° from image center, and fixated more at upper and bottom part of the image^1,2^. Our subjects, however, fixated more uniformly across the image in both correct (Fig. 3a) and error trials (Supplementary Fig. 7a). More fixations were in the upper and bottom part and slightly biased to the left part (Supplementary Fig. 8a, d). There was a fixation hotspot located slightly above the image center (Fig. 3a), which was caused by a large upward bias of the first saccade. The ELM and CTELM models produced a sharp doughnut-shaped distribution of fixation locations, whereas the CCTELM model fixated more uniformly in both correct trials (Fig. 3b–d and Supplementary Fig. 8b, c) and error trials (Supplementary Figs. 7b–d and 8e, f). All the three models fixated more at the upper and bottom part of the image, but unlike the subjects they were not biased toward the left part of the image (Supplementary Fig. 8a, d). Another important difference was that ELM and CTELM models progressively fixated at the outer part of the image as search continues, whereas the subjects’ and CCTELM model’s fixation distance to image center was relatively stable (Fig. 3e–j and Supplementary Fig. 7e–j). Therefore, the goodness-of-fit of the CCTELM model was better and more stable than the ELM and CTELM models (Fig. 3k and Supplementary Fig. 7k).

**Fig. 4:**
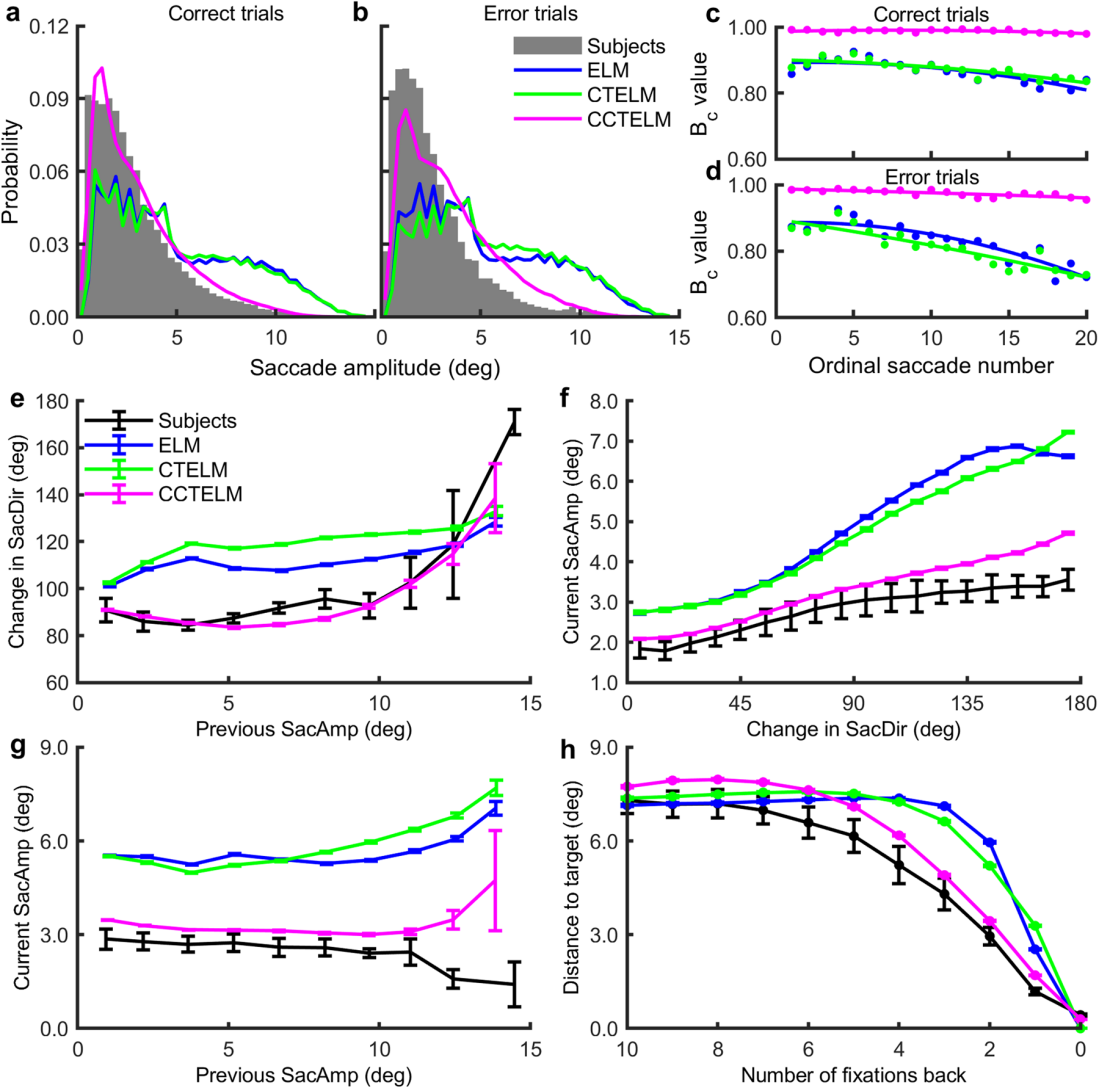
Saccade amplitude distribution of the subjects and models. **a**, **b** Saccade amplitude histogram of all saccades in correct and error trials. **c**, **d** Bhattacharyya coefficient (*B*_c_) between the models’ and subjects’ saccade amplitude distribution of the initial 20 saccades in correct and error trials. Dots represent raw data; curves represent fitted quadratic functions. See Supplementary Fig. 9 for the distribution of raw data at each ordinal position in correct and error trials. **e** Relationship between the first saccade’s amplitude (SacAmp) in two consecutive saccades and the change in saccade direction (SacDir) in all trials. **f** Relationship between the second saccade’s amplitude in two consecutive saccades and the change in saccade direction in all trials. **g** Relationship between the second and the first saccade’s amplitude in two consecutive saccades in all trials. **h** The distance between fixation location and the target location as a function of the number of fixations before correctly finding the target. In panels **e**–**h** error bar represents 95% confidence interval. For experimental data *n* = 6 subjects. The simulation results came from 100,000 independently simulated trials. The distribution of raw data in panels **e**–**h** were shown in Supplementary Fig. 11.

For the saccade amplitude, the two optimal models generated more long saccades, whereas the CCTELM model and human subjects preferred shorter saccades (Fig. 4a, b). The CCTELM model could also consistently fit the saccade amplitude distribution as the search progressed (Fig. 4c, d and Supplementary Fig. 9). Note that the CCTELM model generated more long saccades in the error trials than in the correct trials (Fig. 4a, b) because the last saccade amplitude in the error trials was larger than that in the correct trials (Supplementary Fig. 10). This phenomenon occurred because the error trials were often caused by transient noise in the model, so there was no strong relationship between the saccade amplitude and the number of saccades back before the final response, whereas in the correct trials the fixation locations were gradually attracted to the target location so the saccade amplitude decreased before the final response. In addition, in a sequence of eye movements, the change in the saccade direction after a saccade was usually larger for the ELM and CTELM models than for the CCTELM model and the subjects (Fig. 4e and Supplementary Fig. 11a). Both the subjects and the three models tended to make larger saccades after a larger change in saccade direction, but the slope of the relationship was larger for the ELM and CTELM models than the subjects and the CCTELM model (Fig. 4f and Supplementary Fig. 11b). Moreover, the relationship between saccade amplitude of two consecutive saccades from the CCTELM model was closer to the subjects’ data than the ELM and CTELM models (except for very long saccades) (Fig. 4g and Supplementary Fig. 11c). Owing to the constraints in saccade amplitude, the CCTELM model also approached the target slower than the ELM and CTELM models and was closer to the subjects (Fig. 4h and Supplementary Fig. 11d). In summary, the CCTELM model could better reproduce the subjects’ spatial control of eye movements.

**Fig. 5:**
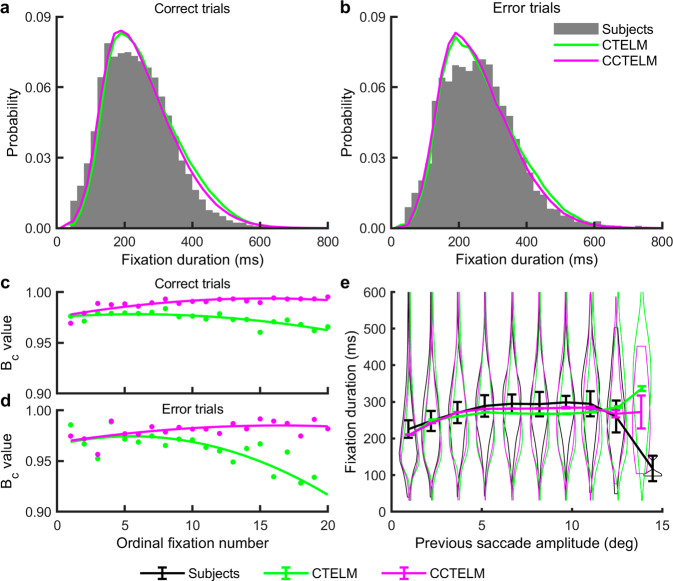
Fixation duration distribution of the subjects and models. **a**, **b** Fixation duration distribution of correct and error trials. The first fixation (at the screen center) and last fixation (when making a response) are excluded. **c**, **d** Bhattacharyya coefficient (*B*_c_) between the models’ and subjects’ fixation duration distributions of the initial 20 fixations after the first saccade in correct and error trials. Dots represent raw data; curves represent fitted quadratic functions. See Supplementary Fig. 12 for the distribution of raw data at each ordinal position in correct and error trials. **e** Relationship between fixation duration and previous saccade amplitude in all trials. Error bar represents 95% confidence interval. For experimental data *n* = 6 subjects. The simulation results came from 100,000 independently simulated trials. The superimposed violin plots represent the distribution of fixation duration in grouped into each bin of previous saccade amplitude.

We then examined how the three models predicted the subjects’ temporal control of eye movements. Both the CTELM and CCTELM models could predict the overall fixation duration distribution (Fig. 5a, b), but the agreement was slightly better as the search progressed in the CCTELM model (Fig. 5c, d and Supplementary Fig. 12). We also checked whether adding saccadic constraints influences the interaction between the spatial and temporal control of eye movements. For the human subjects, as the saccade amplitude increased, the next fixation’s average duration first increased from ~225 ms to ~300 ms and then rapidly decreased to ~120 ms when the saccades were larger than 10° (Fig. 5e). Both the CTELM and CCTELM models could reproduce this relationship when the saccades were smaller than 10° because part of the information at the new fixation location had already been obtained from the previous fixation, so the probability value at the new fixation location was closer to the decision threshold (Supplementary Fig. 13). However, the relationship deviated from the experimental data when the saccade was large (Fig. 5e), which was probably due to the presence of secondary saccades that were not implemented in the model. In summary, the CCTELM model could reproduce subjects’ temporal control of eye movements better than the CTELM model.

### Human visual search performance

The above analysis showed that human eye movement statistics in visual search could be better predicted by a suboptimal strategy with constraints on saccade amplitude, saccade accuracy, and memory capacity. We next compared the search performance of humans and the three models. With the appropriate choice of target detection threshold *θ*_T_ (Supplementary Table 1), the three models’ correct response rate was similar to the subjects in training set (around 88.0%, Table 1), but higher than subjects in the testing set (83.8%, Table 1). This may be caused by individual variability of search strategy and difference in prior training between the two groups of subjects (see discussion).

**Table 1:** Average visual search performance of the two groups of subjects (training and testing set) and three models. *P*(Correct) is the correct response rate.

	*P* (correct)	Median fixation number		Maximum fixation number	
		Correct trials	Error trials	Correct trials	Error trials
Training set	88.09%	7.5	39.3	176	158
Testing set	83.86%	11.5	42.5	181	428
ELM	87.57%	7	6	43	34
CTELM	88.15%	7	7	40	37
CCTELM	87.40%	8	8	125	102

**Fig. 6:**
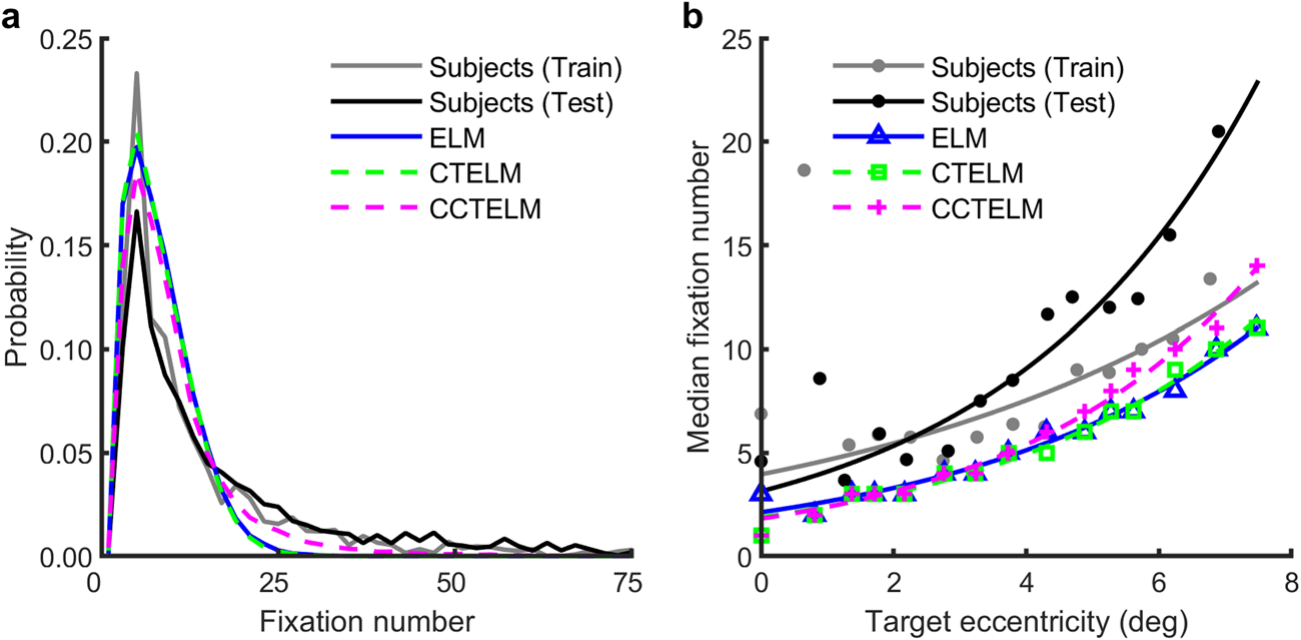
Subjects and the three models’ search performances. **a** The distribution of the number of fixations needed to find the target in all correct trials of the subjects and three models. **b** Average median number of fixations to correctly find the target as a function of the median target eccentricity from the image center. The median fixation numbers were first calculated for each subject then averaged across subjects. Dots are raw data and curves are fitted exponential function. The two gray and black dots within target eccentricity range 0.6–0.9° were treated as outlier when fitting the function.

In terms of the distribution of fixation numbers needed to correctly find the target, the subjects’ and the three models’ data had a similar peak, but the subjects and the CCTELM model had a higher probability of fixating more than 20 times (Fig. 6a). The median fixation number in the correct trials was similar across the three models and subjects in the training set (7–8 fixations), but subjects in the testing set were 3–4 fixations slower (Table 1). This discrepancy was not very large considering that the mean fixation duration was ~250 ms. However, both groups of subjects were much slower than the three models in error trials (Table 1), and possible reasons will be discussed in the discussion section. With respect to fixation number as a function of target eccentricity, subjects in the training set performed closer to the CCTELM model except that they were much slower when target eccentricity was about 0.6° (Fig. 6b). Subjects in the testing set also searched slower when target was distant (Fig. 6b). In summary, the subjects’ search speeds were suboptimal, but they could still keep a relatively low median fixation number.

As in previous studies^1–3^, we measured the visibility map with the target location cue in the detection task; one may question the validity of using it in the visual search task when there is no target cue. We also measured the visibility map without the target location cue in four subjects (Supplementary Fig. 14) and found that the optimal model searched slower than the subjects (Supplementary Fig. 15). Therefore, in theory, visibility map measured without target location cue does not allow human-level search performance. In the discussion, we also discuss our belief that the visibility map measured without the target location cue was probably not an accurate measure of the actual visibility map in visual search.

### The effects of memory and saccadic constraints

**Fig. 7:**
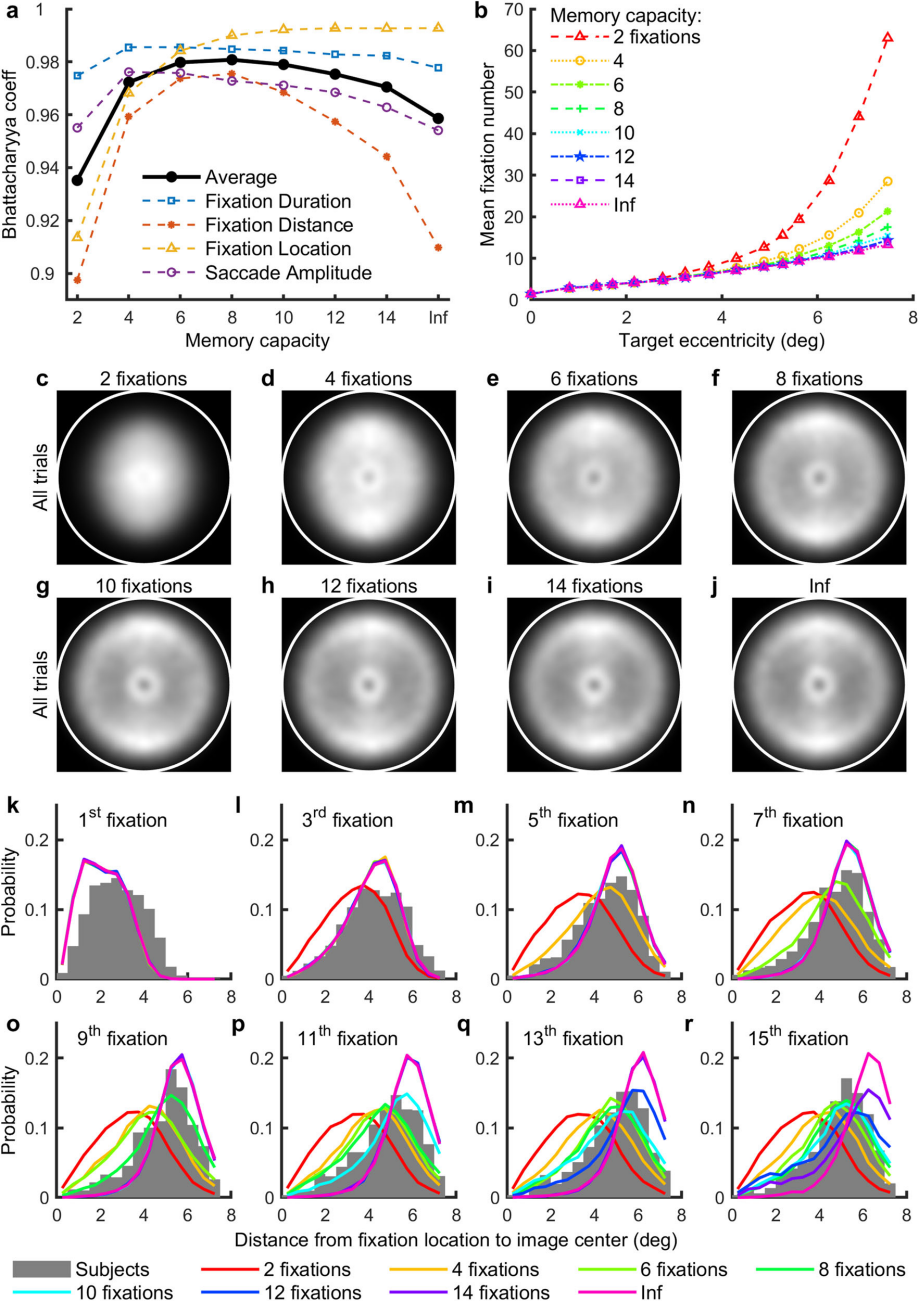
Effect of memory capacity on the CCTELM model’s behavior. **a** Effect of memory capacity on the goodness of fit to training data. For fixation duration, fixation distance (to screen center) and saccade amplitude, we first align sequences of eye movements at start of the trial and calculated the initial 20 histograms of these metrics from all (both correct and error) trials. Then we calculated the 20 Bhattacharyya coefficients (*B*_c_) at each ordinal position and averaged them together to obtain a single value for each memory capacity per metric. For fixation location we calculated the overall 2-dimensional histogram from all trials, smoothed by a Gaussian window with SD = 0.35° (15 pixels), and calculated the *B*_c_ for each memory capacity. The thick black line is the average of the four colored thin dashed lines. “Inf” represents unlimited memory capacity. **b** Effect of memory capacity on the mean number of fixations to correctly find the target at different eccentricities of the CCTELM model. The data in panels **a** and **b** came from the CCTELM model whose parameters were fit assuming unlimited memory but evaluated under different memory capacities. **c**–**j** Effect of memory capacity on fixation location distribution (lighter means higher density). White circle is the boundary of search field. **k**–**r** Effect of memory capacity on fixation distance distribution to image center within the initial 15 fixations after the first saccade. The gray bars were subjects’ data in the training set, and lines with different colors in each panel represent model result with different memory capacity.

We have shown that the CCTELM model agreed better with human visual search eye movement metrics. The remaining questions were how we estimated the memory capacity, and why these constraints improved the model’s prediction. We have varied the CCTELM model’s memory capacity and examined the goodness-of-fit to the training set. Figure 7a shows that the distributions of fixation distances to screen center and fixation location were most sensitive to memory capacity, and the distributions of fixation duration and saccade amplitude were less sensitive unless at extreme values. Model with a low memory capacity fixated on the central part of the image too often (Fig. 7c–j), and model with a large memory capacity fixated more on the outer part of the image as search progressed (Fig. 7k–r). On average, the matching with the experimental data peaked when the memory capacity was 8 fixations (Fig. 7a). Decreasing the memory capacity to 6–8 fixations did not greatly affect the mean fixation number needed to find the target, and the discrepancy was only present when the target was distant (Fig. 7b). In summary, a model with memory capacity of 8 fixations could better predict the experiment data while the search performance is only slightly affected. Therefore, we choose 8 fixations as the memory capacity of CCTELM model.

**Fig. 8:**
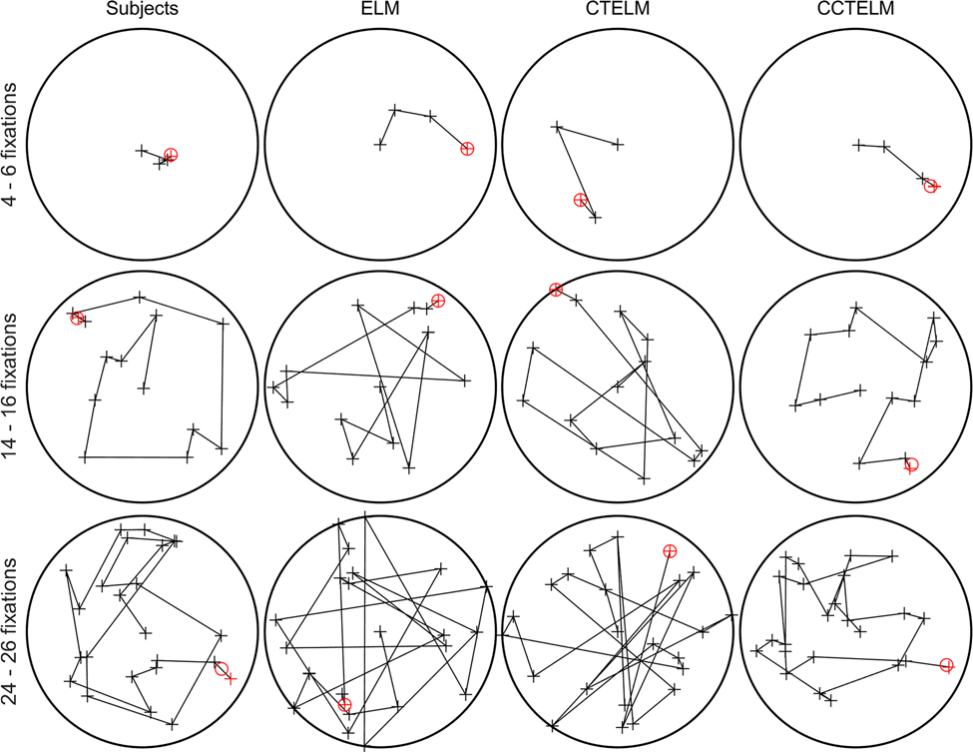
Example scan path of the subjects and models. Scan paths on each row have similar fixation number shown at the left side. The search starts from image center. Black circle represents the image boundary, cross represents a fixation, and the line between two crosses represents a saccade. The final fixation to report the target location is labeled in red. The red circle shows the true target location.

We further checked the scan path of humans and models to understand the effect of saccade amplitude penalty function. Subjectively, the scan path looked similar when the target was close and number of fixations was small, but difference emerged when the number of fixations was larger (Fig. 8). The ELM and CTELM models chose the location that maximize the expected information gain as the next fixation location. In general, one can get most information by fixating at novel regions and avoid previously fixated regions. Therefore, both the ELM and CTELM models tended to avoid image center (where the search starts) and jumped between opposite sides of the search field (Fig. 8), producing many long saccades (Fig. 4a, b) and larger change in saccade direction (Supplementary Fig. 16). Adding constraint on saccade amplitude prevent this behavior and forced the model to make series of small saccades around the image to reach the other side of the image (Fig. 8), so the change in saccade direction decreased and was closer to humans’ behavior (Supplementary Fig. 16).

Saccadic inaccuracy was introduced into the CCTELM model to solve a subtle technical issue. Without saccadic inaccuracy, the ELM and CTELM models could only fixate at the 400 predefined locations. As these locations were uniformly sampled inside the search field, the distances between two adjacent locations were similar (Supplementary Fig. 5a). This made the distribution of saccade amplitudes “discrete-like” when the amplitude was small (Supplementary Fig. 5b). Since the CCTELM model produced many short saccades, these saccades would be grouped into a single histogram bin when plotting the distribution of saccade amplitudes. Moreover, constraining the fixation locations to a limited predefined set of locations introduced the potential question of whether the model’s behavior depends on the number of predefined locations. In this research, the minimum distance between adjacent locations was ~0.7° and the minimum SD of saccade landing position was ~0.4° (Supplementary Fig. 6b), so the CCTELM model could fixate at any location within the search field. Therefore, adding saccadic inaccuracy mitigates the dependency on predefined locations, and smooths the distribution of saccade amplitudes.

**Fig. 9:**
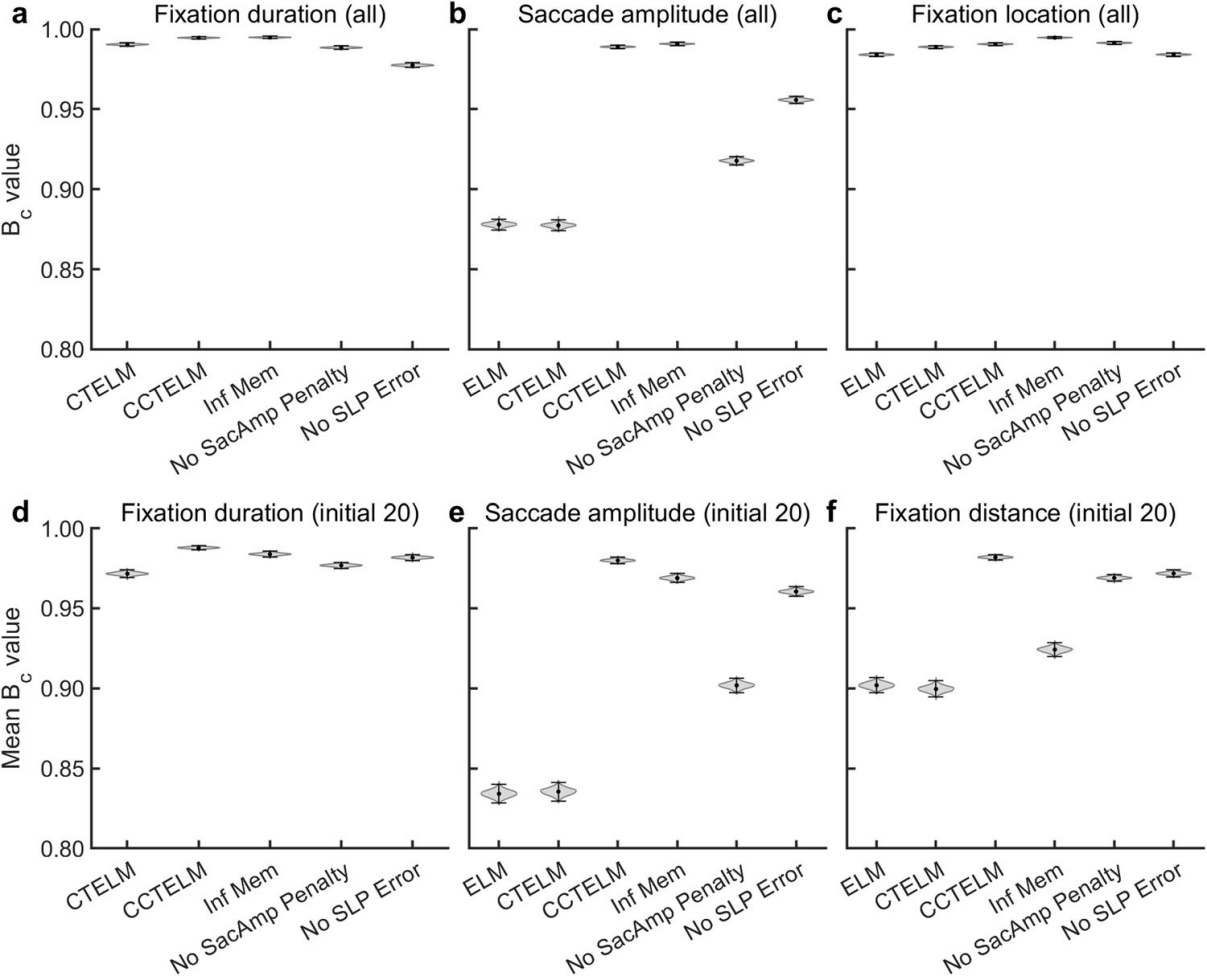
The effect of three constraints in the CCTELM model on the goodness-of-fit of different eye movement metrics. In all panels, “CCTELM” represents the full CCTELM model; “Inf Mem” represents the CCTELM model with infinite memory capacity; “No SacAmp Penalty” represents the CCTELM model without saccade amplitude penalty function; “No SLP Error” represents the CCTELM model without saccade landing position offset and variance. The last three models were evaluated using the same parameters as the CCTELM model. **a**–**c** Bhattacharyya coefficient (*B*_c_) between the subjects’ and models’ overall fixation duration (**a**), saccade amplitude (**b**), and fixation location (**c**) distributions. **d**–**f** Average *B*_c_ between the subjects’ and models’ fixation duration (**d**), saccade amplitude (**e**), and fixation distance to image center (**f**) distributions of the initial 20 fixations or saccades. The average *B*_c_ was obtained by first calculating 20 *B*_c_ for each of the initial 20 fixations or saccades after the start of a trial (thus obtaining 20 *B*_c_ values at each ordinal position in eye movement sequences), and then averaging the 20 *B*_c_ values. The 99% confidence interval (shown in error bar) was calculated by bootstrapping the experimental and simulation data for *n* = 2000 times and then multiplying the SD of all *B*_c_ by 2.576. The violin plot represents the distribution of *B*_c_ from all bootstrapping trials.

To better see the relative importance of the three constraints to the improvement of model’s goodness-of-fit, we compared the three models and the CCTELM model without each of the three constraints (using the same parameter values) to experimental data. The *B*_c_ of the ELM and CTELM models were indeed lower than those of the CCTELM model in both the spatial and temporal aspects of eye movement statistics (Fig. 9). The three constraints had a relatively small effect on the *B*_c_ of the fixation duration and fixation location distribution (Fig. 9a, c, d). Removing the saccade amplitude penalty function had a large impact on the *B*_c_ of the saccade amplitude distribution, and removing the saccade inaccuracy had a smaller impact (Fig. 9b, e). Removing the memory capacity limitation mostly affect the *B*_c_ of the distribution of fixation distance to screen center (Fig. 9f). In summary, the constraints on saccade amplitude and memory capacity improved the CCTELM model the most, and the constraint on saccade accuracy improved the model to a lesser degree.

### Generality of the CCTELM model

Since measuring the visibility map requires multiple experiment sessions, it is the principal limitation to increasing the sample size. In addition, the visibility map is also highly specific to the properties of the target and background used in the experiment. One may therefore wonder how generalizable the model is. We compared our model (with the same parameter values) with the data from a slightly different visual search experiment. In that experiment, we used the same background and target image, but the target contrast was fixed to 0.15 (higher than the current experiment). Seven subjects were recruited (four were new subjects), and we did not measure the visibility map. We found that there was still considerable overlap of the model’s and this new set of subjects’ eye movement metrics, except that subjects’ distribution of fixation location had a more pronounced hotspot slightly above the image center, and was more uniformly distributed elsewhere (Supplementary Fig. 17). This showed that the CCTELM model could potentially be generalized to a larger set of subjects, even for an experiment with different target contrasts.

## Discussion

In this study, the comparison of eye movement statistics between humans and both the ELM and CTELM models showed clear differences in the distribution of fixation location, fixation distance to image center, saccade amplitude, and the dependency between successive eye movements. The CCTELM model further suggested that the discrepancy was mainly due to constraints on saccade amplitude, saccade accuracy and memory capacity. Therefore, if we consider the degradation of visibility in peripheral vision as the only limitation to visual search, the subjects clearly employed a suboptimal eye movement strategy to find the target.

However, this suboptimal strategy did not greatly increase the median fixation numbers. Considering the physiological meanings of the constraints in CCTELM model, we think that humans may be balancing task performance and costs when making eye movements to search for target. If humans try to use the optimal eye movement strategy to fully use the visibility map, they will need to make more long saccades, which means more time spent on saccades^10^, more subsequent corrective saccades because of decreased landing accuracy^9^, and stronger saccadic suppression during the saccade^11^. They also need to maintain a long history of previous fixation in memory, which will also increase the cognitive load. Choosing a suboptimal eye movement strategy that reduces these costs while maintaining a high search performance should, therefore, be a better solution.

Our results were different from a previous similar experiment, which showed that human eye movement statistics were consistent with the optimal model^1,2^. This could be caused by both individual variability of visual search strategies and the difference of the amount in training prior to the visual search experiment. Previous results^2^ came from two subjects on whom the target visibility had been measured with more trials at more retinal locations, and had performed more visual search trials with more target visibility levels, so it is possible that previous results were the behavior of two well-trained subjects whose performance was closer to the optimal model. In our study, we recruited more subjects, so because of time limitations we could not obtain a very densely sampled visibility map and perform many visual search trials, and we did not measure the full temporal course of visibility on some subjects in the testing set before the visual search experiment. This may cause the behavioral differences between different groups of subjects, but the current results may be more generalizable to a larger pool of subjects.

Several other studies have also shown that human eye movements are suboptimal when optimally performing a task required effortful computation and careful planning^4–6^, so subjects may simply follow good heuristics when searching in these noisy images. The advantage of this strategy is that the computational cost of saccade target selection may be further reduced to selecting random location from a heuristic distribution^6^, as even the relatively computationally efficient ELM rule requires convolving two maps across the visual field in every fixation. The heuristics may be good enough but not strictly optimal for this particular task. From this perspective, the subjects may also be closer to optimal balance between search performance and the cost to perform the task.

The CCTELM model controlled fixation duration by a drift-diffusion process based on experimentally measured temporal course of visibility and an artificial collapsing decision threshold. Our approach shared some similarities to some previous models, which controlled fixation duration collectively by an autonomous timer and modulation by processing demand from the current fixation^21,22,24^. From this viewpoint, the CCTELM model implemented the autonomous timer as the collapsing threshold, and modulation from the current fixation as the drift-diffusion process. Fixation will be prolonged if the model cannot determine whether the current fixation location has a target because the probability value at the fixation location will not decrease quickly. However, our approach differed from previous models in that the diffusion process was constructed from experiment data, thus being more biologically realistic, and it is based on probability values so it can be incorporated into the ideal searcher theory. The introduction of a collapsing threshold may seem arbitrary, but its existence, though still controversial, has received certain experimental support^33,34^, and it allows human to trade-off accuracy for speed if the current visual information remains ambiguous about target location. Besides, though the CCTELM model seemed to control the spatial and temporal aspects of eye movement separately (Fig. 2), the relationship between previous saccade amplitude and the next fixation duration (Fig. 5e) showed that the two aspects were not completely independent because they both depended on the posterior probability map.

An interesting discovery is that the subjects’ eye movement statistics predict the memory capacity in visual search. Similar capacity value has also been used in some models of scene viewing^35^, but many models of visual search still ignore this memory limitation. We found that the memory capacity had a considerable effect on the model’s eye movement statistics and its agreement to experimental data, so it may be worthwhile for future simulation studies to consider this factor. Memory helps not only in identifying the target, but also in rejecting regions without the target so that the model had a higher chance to meet the target in the future. If the memory capacity is too small, the model will quickly forget information near the image center (where the search starts), and by the ELM rule, the model will tend to place subsequent fixations near the image center to gain maximal information (fixating near the image boundary causes part of the visibility map to be wasted). Conversely, if the memory capacity is too large, the model will not forget the information near the image center, and by the ELM rule the model will tend to search the outer unexplored part of the image to gain maximal information. It is mainly the combination of these two effects that helps us to estimate the memory capacity.

The type of short-term memory that we modeled is similar to the fragile visual short-term memory (FVSTM)^36,37^. FVSTM is thought to be an intermediate stage between iconic visual memory and visual working memory. It has a high capacity of ~5–15 items, lasts for at least 4 s^36^ and has been shown to be tied to the location of the original visual stimulus^37^, making it particularly suitable for integrating information across multiple fixations to generate a posterior probability map. However, the exact memory capacity in visual search tasks remains controversial. There are reports indicating that visual search has a memory of 3–4 items^38^, 7−9 items^39,40^, and >10 items^41^. The predicted memory capacity of eight fixations of the CCTELM model lies within the range of these reported results and thus may serve as additional evidence in this debate. However, the exact memory capacity requires future investigations and may serve as a test of our model.

Visual search is a complex behavior that involves multiple brain regions, and the experiment and computational methods in this study are still not perfect. Measuring the visibility map requires a large number of trials, which limits the sample size and the generality of the visibility map of this study. In addition, the visibility map was measured with target location cue, but this was not the true situation in the visual search experiment. We think that the subjects’ actual visibility map in visual search experiment was somewhere between the visibility map measured with and without the target location cue, because previous studies have shown that visual sensitivity will increase at future fixation locations in a sequence of eye movements, even to the level comparable to the visual sensitivity measured when the target location is cued^42,43^. However, the exact visibility map is difficult to measure because it depends on the planned future eye movement sequence.

The CCTELM model was relatively simple compared to the real brain. For example, the model either kept information from a fixation or completely forgot it, whereas in humans, memory may gradually decay over time^44^, which was considered in a previous model of scene viewing^35^. Multiple types of memory may be involved in visual search^45,46^, but this was not reflected in the current model. In addition, humans may plan multiple fixations ahead during a fixation^47^, whereas our model plans only one fixation ahead. We also did not include the generation of secondary saccades in the model. Humans tend to undershoot after a long saccade and then make a short corrective secondary saccade to the original saccade target, which may explain why the mean saccade amplitude decreased after a long saccade (Fig. 4g). From previous data^19^, we also found that the mean fixation duration before secondary saccades was ~116 ms, which is very close to the mean fixation duration after a saccade of ~15° in our data. However, the previous study measured the frequency of secondary saccades only in a visually guided saccade task^19^. Therefore, since we did not know the frequency of secondary saccades in eye movement sequence during this natural visual search task, we did not consider secondary saccades in the model.

Although the CCTELM model is suboptimal, the median search speed was still faster than subjects in correct trials. This finding suggests that the model did not capture all the suboptimalities in the human eye movement strategy. One possible missing suboptimality is the bias in choosing the fixation location. An obvious bias of the subjects’ fixation location was the hotspot located above the image center. The hotspot was caused by a large upward bias in the first saccade from some subjects, but the reason for this behavior is unknown. Since the target was randomly embedded inside the image and there was no spatial bias in the statistical features of the search images, such bias in choosing fixation location may influence subjects’ search speeds. For example, if the target was located just below the image center, the upward bias in the first saccade may cause the subjects to miss the target, and this might explain why subjects searched surprisingly slow when the target was about 0.6–0.9° away from the image center.

Another factor that may limit subjects’ search performance is the trade-off between response accuracy and search speed. In the CCTELM model a response was made whenever the posterior probability at the current fixation location exceeded the target detection threshold, but in humans, this process may be much more complex. For example, when there were multiple locations similar to the target, subjects who emphasized more on response accuracy may actively compare them, which could greatly decrease the search speed. Besides, when the target was located near the image center, subjects may note it initially, but still intentionally scan across the image to confirm the target location. The individual variability on this trade-off may also explain the individual variability of search speed besides the different amount of training mentioned previously.

Finally, none of the three models generated as many fixations as subjects displayed in the error trials. One possible reason is that we used dynamic noise instead of static noise in the actual experiment. We think that there are at least two reasons that cause humans to make visual search errors: (1) A local feature in the noisy background image was very similar to the target and caused misidentification; and (2) A local feature in the noisy background image made the target too hard to identify. Both reasons are related to the static noise in the stimulus image. Particularly in the second case, subjects may search repeatedly before giving up, which might explain why the fixation numbers are much larger in the error trials than in the correct trials. However, since we used dynamic noise in the model, the second scenario above will not occur because the noise in each fixation is independent, and the probability of repeatedly obtaining a high noise level at the target location is very low. Therefore, the model is unlikely to generate a large number of fixations in the error trials.

## Methods

### Human subjects

Ten student volunteers (six males, aged 19–30 years) participated in the experiment for monetary compensation. All subjects had no neurological or psychological disease history and had normal or corrected-to-normal vision. This study was approved by the Ethics Committee of the School of Life Sciences at Fudan University, and all subjects provided written informed consent.

### Apparatus

Stimuli were presented on a 24.5-inch BenQ ZOWIE XL2540 LCD monitor at a resolution of 1920 × 1080 and a refresh rate of 240 Hz. Subjects were seated 70 cm in front of the display with their heads fixed by a forehead and chin rest. Stimuli were generated and presented using MATLAB (MathWorks) and the Psychophysics Toolbox^48–50^ running on a Windows 7 (Microsoft) machine. Eye movements of both eyes were recorded at 2000 Hz with a TRACKPixx3 eye-tracker (Vpixx Technologies, Canada). During each laboratory visit, subjects first performed a 13-point calibration routine until the average test-retest measurement error of both eyes fell below 0.5°. Recalibration was performed during experiment whenever the eye-tracking accuracy failed to meet the requirements of the experiment (described below).

### Target and background image

The target was a six cycle-per-degree Gabor with a diameter of 0.3°, oriented 45° counterclockwise from the vertical, and windowed by a symmetrical raised cosine (half-height width was one cycle of the Gabor). The background image was a circular naturalistic noise image (1/*f*^*2*^ power spectrum) placed at the screen center with a diameter of 15° and root-mean-squared (RMS) contrast of 0.2 (Fig. 1a). The RMS contrast was the SD of the pixel gray value divided by the mean. The area outside the background image was set to the mean gray value of the image (0.5). The background images used in each trial of detection task and visual search task were randomly picked from a database of 1000 independently generated images.

### Selecting target RMS contrast

In this experiment, we measured the relationship between foveal target visibility and target RMS contrast and selected the target contrast with visibility = 3.0. Each participant had a unique target contrast and was used for all subsequent experiments. The goal was to normalize the target detection difficulty across subjects.

At the start of each trial, a black cross and stroked circle appeared at screen center, indicating the fixation and target location. The remaining display area was set to 0.5 gray value. Subjects began a trial by fixating within 1° from the screen center and pressing a button. The cross and circle then disappeared for 200−400 ms (chosen randomly), followed by two 250 ms intervals of the stimulus image separated by a 500 ms gray interval. The two stimulus images had the same noise background, but a randomly chosen one had a target at the center. The subjects then chose the interval that contained the target with a keyboard. A trial was aborted if the participant fixated more than 1° away from the screen center at any time during the trial. This section contained 4−5 levels of target contrast (ranging from 0.03 to 0.12), and each level had 200 trials. Trials with different target contrasts were randomly interleaved.

We first calculated the hit rate and correct rejection rate of each target contrast level from behavior data. The hit (or correct rejection) rate was defined as the number of trials that target appeared in the first (or second) interval, and the participant made a correct response, divided by the total number of trials that target appeared in the first (or second) interval. The relationships between the hit/correct rejection rate and target RMS contrast *C* were then separately fit with a Weibull function:

1 $${f\left( C \right) = 0.5 + 0.5 \times \left\{ {1 - \exp \left[ { - \left( {\frac{C}{{C_{\mathrm{T}}}}} \right)^s} \right]} \right\}}$$

The threshold parameter *C*_T_ and steepness parameter *s* were estimated by maximum-likelihood methods implemented in the Palamedes Toolbox^51^. The relationship between the foveal target visibility and target RMS contrast could then be estimated by signal detection theory^52^:

2 $${d^\prime \left( C \right) = \frac{{z\left[ {f_{\mathrm{H}}\left( C \right)} \right] - z\left[ {1 - f_{{\mathrm{CR}}}\left( C \right)} \right]}}{{\sqrt 2 }}}$$

Here, *z*(*x*) is the inverse standard normal cumulative distribution function. *f*_H_(*C*) and *f*_CR_(*C*) are the estimated hit and correct rejection rates given by Eq. (1). We could then obtain the target contrast that made *d*′(*C*) = 3.0.

### Detection task

The goal of the detection task was to measure the relationship between the stimulus presentation time and target visibility along the four cardinal directions in the search field. The task had three versions. Four subjects participated in the first version, four subjects participated in the second version (one participated in both versions), and three subjects participated in the third version. The subjects completed the task in multiple laboratory visits within 2 months. The procedure was the same as the experiment to select the target RMS contrast, except that we varied both the stimulus duration and the target location. Before the start of the formal experiment, they practiced for 25–100 trials for performance stabilizations. The subjects received feedback about their performance only in the practice trials.

In the first version of the experiment, the subjects were required to fixate on the screen center and identify which of the two stimulus intervals contained the target at the cued location. The experiment contained five blocks (the four cardinal directions plus the screen center). In each block, we chose 3 − 4 different target locations (except when the target was in the center of the screen) and 4 − 5 levels of stimulus presentation time. The tested target locations were determined for each subject by a pilot experiment. In the pilot experiment, we found the smallest target eccentricity that makes the detection performance correspond to roughly the chance level (50–60% correct rate after practicing), and this eccentricity was the maximum target eccentricity used in the formal experiment. In the formal experiment, when the target appeared at the screen center, the stimulus duration was chosen from 4 to 200 ms, and each level was tested for 100 trials. When the target appeared at peripheral locations, the stimulus duration was chosen from 50 to 700 ms, and each stimulus length and target location combination was tested for 50 trials. This version involved ~5000 trials.

In the second version of the experiment, the target location cue was not shown, and in each trial, the target location and stimulus presentation time were randomly chosen from all possible combinations in the first version of the experiment. This version involved ~5000 trials. The results are shown in Supplementary Fig. 14, but they are not used in the results of the main text.

The third version was the same as the first version except that there was only one level of stimulus presentation time (250 ms). It was used to measure the visibility map efficiently and obtain enough training to prepare for the visual search experiment, so the data from this version were not used in this manuscript. This version involved 1500 trials.

To calculate the visibility map measured with or without the target location cue, the behavior data from different subjects in each version were first pooled together, and the target visibility at each location for each stimulus duration level was then calculated by Eq. (2). Note that since the subjects had different visibility maps, some locations were sampled from multiple subjects, while others were sampled from a single subject. The points that were sampled from more subjects were given proportionally larger weights when fitting the visibility map function (see below).

### Evidence accumulation model

We extended the previously proposed template response model^1–3^ to visual stimulus at each visual field location to a drift-diffusion process *W*_*t*_, and was later used in the continuous-time eye movement model. Suppose the visual evidence ∆*W* generated at one location within a short time interval ∆*t* is independently drawn from a normal distribution. If the target appears at this location, the distribution is *N*(∆*t*, σ^2^∆*t*), otherwise *N*( − ∆*t*, σ^2^∆*t*). The value of σ controls target visibility at this location. If the evidence at each time step ∆*t* is integrated perfectly, then the total evidence at time *T* = *n* ∙ ∆*t* follows the distribution of the sum of *n* normal random variables:

3 $$\left\{ {\begin{array}{*{20}{l}} {N(T,\sigma ^2T),} \hfill & {{\mathrm{if}}\,{\mathrm{target}}\,{\mathrm{exists}}} \hfill \\ {N( - T,\sigma ^2T),} \hfill & {{\mathrm{if}}\,{\mathrm{no}}\,{\mathrm{target}}\,{\mathrm{exists}}} \hfill \end{array}} \right.$$

According to signal detection theory^52^, the evolution of target visibility *d*′ over time is:

4 $${d^\prime \left( T \right) = \frac{{T - \left( { - T} \right)}}{{\sqrt {\sigma ^2T} }} = \frac{{2\sqrt T }}{\sigma }}$$

Equation (4) implies that target visibility will increase to infinity at any retina location given unlimited amount of viewing time, which is impossible. This problem can be resolved if we assume that the weight *w* of previous evidence decays exponentially by a speed parameter *k* over time:

5 $$\begin{array}{*{20}{c}} {w\left( t \right) = \exp \left( { - k \cdot t} \right)} \end{array}$$

Suppose the total accumulated evidence at one location from 0 to the *i*^th^ time step is *W*_*i*_ (*W*_0_ = 0), then by the (*i* + 1)^th^ time step, the total accumulated evidence is:

6 $$\begin{array}{*{20}{c}} {W_{i + 1} = {\Delta}W + W_i \cdot w\left( {{\Delta}t} \right)} \end{array}$$

Then the total acquired evidence *W*_*n*_ at time *T* = *n* ∙ ∆*t* follows the distributions:

7 $$\left\{ {\begin{array}{*{20}{l}} {N\left( {{\Delta}t \cdot \mathop {\sum }\limits_{i = 0}^{n - 1} w\left( {i \cdot {\Delta}t} \right),\sigma ^2{\Delta}t \cdot \mathop {\sum }\limits_{i = 0}^{n - 1} w\left( {i \cdot {\Delta}t} \right)^2} \right),} \hfill & {{\mathrm{if}}\,{\mathrm{target}}\,{\mathrm{exists}}} \hfill \\ {N\left( { - {\Delta}t \cdot \mathop {\sum }\limits_{i = 0}^{n - 1} w\left( {i \cdot {\Delta}t} \right),\sigma ^2{\Delta}t \cdot \mathop {\sum }\limits_{i = 0}^{n - 1} w\left( {i \cdot {\Delta}t} \right)^2} \right),} \hfill & {{\mathrm{if}}\,{\mathrm{no}}\,{\mathrm{target}}\,{\mathrm{exists}}} \hfill \end{array}} \right.$$

Note that:

8 $$\begin{array}{*{20}{c}} {\mathop {{\lim }}\limits_{{\Delta}t \to 0} \left[ {{\Delta}t \cdot \mathop {\sum}\limits_{i = 0}^{n - 1} w \left( {i \cdot {\Delta}t} \right)} \right] = \mathop {\int}\limits_0^T w \left( t \right)dt = \frac{{1 - \exp \left( { - kT} \right)}}{k}} \end{array}$$

9 $$\begin{array}{*{20}{c}} {\mathop {{\lim }}\limits_{{\Delta}t \to 0} \left[ {{\Delta}t \cdot \mathop {\sum}\limits_{i = 0}^{n - 1} w \left( {i \cdot {\Delta}t} \right)^2} \right] = \mathop {\int}\limits_0^T w \left( t \right)^2dt = \frac{{1 - \exp \left( { - 2kT} \right)}}{{2k}}} \end{array}$$

Substituting Eqs. (8) and (9) into Eq. (7), the evolution of target visibility over time can be formulated as:

10 $${d^\prime \left( T \right) = a\sqrt {\frac{{1 - \exp \left( { - kT} \right)}}{{k\left[ {1 + \exp \left( { - kT} \right)} \right]}}} ,{\mathrm{where}}\,a = \frac{4}{{\sqrt 2 \sigma }}}$$

We first fit Eq. (10) to the temporal course of target visibility at each measured retinal location (Supplementary Fig. 2) and got the values of parameters *a* and *k* at different locations (*x*, *y*) relative to the fixation location (0, 0). We found that the values of these two parameters decay exponentially from fixation location (Supplementary Fig. 3). Considering the asymmetry between horizontal and vertical visibility, we fit the values of *a* and *k* by:

11 $$\begin{array}{*{20}{c}} {a\left( {x,y} \right) = p_1 \cdot \exp \left( { - p_2 \cdot \sqrt {x^2 + p_5 \cdot y^2} } \right)} \end{array}$$

12 $$\begin{array}{*{20}{c}} {k\left( {x,y} \right) = p_3 \cdot \exp \left( { - p_4 \cdot \sqrt {x^2 + p_5 \cdot y^2} } \right)} \end{array}$$

Equations (10)–(12) together describe the change of visibility map over space and time. The parameters *p*_1_, *p*_2_, *p*_3_, *p*_4_, *p*_5_ in these equations were estimated by minimizing the MSE between the calculated and measured visibility data using the GlobalSearch algorithm in the Global Optimization Toolbox of MATLAB. Since the pooled experimental data at each measured location may come from a different number of subjects, to make each subject’s contribution to the pooled visibility map roughly equal, the points that were sampled from more subjects were given proportionally larger weights when fitting the visibility map function.

### Visual search task

In this experiment, subjects searched for the target within the background noise image as fast as possible while trying to maximize the response accuracy. They were informed that the target locations were sampled uniformly within the circular noise region of the image. The subjects began each trial by fixating within 0.8° away from a cross displayed in the center of the screen (checked by the eye-tracker) and pressing a button. Then, the cross disappeared for a random interval (0.2–0.3 s), and the search image appeared (Supplementary Fig. 1c). No limits were imposed on the searching time. When the subjects found the target, they fixated on the target and pressed the keyboard. The final fixation location and the target’s true location were then shown. If the fixation error was less than 1°, we labeled the response as correct. We recalibrated the eye-tracker if subjects could not proceed at the start of a trial, or if subjects had found the correct target location, but the recorded final fixation location was more than 0.8° away from the target location. On average, subjects recalibrated 3.3 times (range of 0.5–5.5 times) per 100 trials. All 10 subjects completed this experiment, but the numbers of trials were different (200 − 500) depending on their available time (Supplementary Table 2).

To examine the generality of the visual search model, seven subjects (four were new subjects) participated in an extended visual search task. The experimental procedure and the background and target image were the same, but the target RMS contrast was fixed to 0.15. Each subject completed 100–300 trials depending on the available time. The data from this experiment were used only as the testing set in Supplementary Fig. 17.

### Eye-tracking data analysis

Eye-tracking data were analyzed by the EYE-EEG extension^53^ of the EEGLAB toolbox^54^. We used an adaptive velocity-based algorithm^55^ to classify eye movements into saccades, fixations and blinks. Fixations separated by blinks were recognized as two fixations. To address intermittent noise in the data, we used the minimum instantaneous eye movement velocity in the event classification algorithm. In practice, we found that this method can effectively handle noisy data from a single eye. In all the subjects, only two trials with unreliable recordings in both eyes were rejected. Data from the four subjects in the first version of the detection task served as the training set to fit the parameters of the eye movement model, and data from the remaining 6 subjects served as the testing set (Supplementary Table 2). The training set contained 21,439 fixations and 19,645 saccades from 1190 trials, and the testing set contained 44,320 fixations and 40,190 saccades from 1958 trials (Supplementary Table 2). The data in the extended visual search experiment contained 23,477 fixations and 21,338 saccades from 1502 trials.

To quantify the noise level of the eye movement data, we calculated the SD of the data samples and the RMS of inter-sample angular distances for each fixation for each eye of each subject, then averaged across the two eyes. These two metrics were calculated as:

13 $$\begin{array}{*{20}{c}} {{\mathrm{SD}}_{{\mathrm{fix}}} = \sqrt {\frac{{\mathop {\sum }\nolimits_{i = 1}^{N_{{\mathrm{sample}}}} \left[ {\left( {x_i - \bar x} \right)^2 + \left( {y_i - \bar y} \right)^2} \right]}}{{N_{{\mathrm{sample}}}}}} } \end{array}$$

14 $$\begin{array}{*{20}{c}} {{\mathrm{RMS}}_{{\mathrm{fix}}} = \sqrt {\frac{{\mathop {\sum }\nolimits_{i = 1}^{N_{{\mathrm{sample}}} - 1} \left[ {\left( {x_{i + 1} - x_i} \right)^2 + \left( {y_{i + 1} - y_i} \right)^2} \right]}}{{N_{{\mathrm{sample}}} - 1}}} } \end{array},$$

where *N*_sample_ is the number of data samples in one fixation, (*x*_*i*_, *y*_*i*_) is the horizontal and vertical gaze location of the *i*^th^ sample of one eye, and $\left( {\bar x,\bar y} \right)$ is the average gaze location of one fixation of one eye.

When summarizing fixation durations, we excluded the first (when fixating at screen center) and the last fixations (when making response) to avoid the effects of the anticipation and motion preparation on the fixation duration. When summarizing fixation locations, we excluded the first fixation. We did not discard any fixations when summarizing the fixation number needed to find the target in each trial. As the number of eye movement events of each subject was imbalanced (Supplementary Table 2), the eye movement metrics were first summarized for each subject and then averaged across subjects.

### The entropy-limit minimization (ELM) model

We used the ELM model as our baseline model for replication of previous study^1^. The model could only fixate at 400 locations uniformly sampled inside the search field (Supplementary Fig. 5a). We set the fixation duration to 250 ms and evaluated the visibility map using Eq. (10). The visual information $W_{i,L_F}$ obtained at location *i* (*i* = 1⋯*n*, *n* = 400) during the *F*^th^ fixation at location *L*_*F*_ was sampled from normal distribution $N\left( {0.5,1/d_{i,L_F}^{\prime 2}} \right)$ if the target was at this location, otherwise $N\left( { - 0.5,1/d_{i,L_F}^{\prime 2}} \right)$. The ELM model did not consider fixation duration, so the visual information at each location was sampled once per fixation. The posterior probability map of target location was calculated from information gathered in all previous fixations (unlimited memory) according to Bayesian theory:

15 $$\begin{array}{*{20}{c}} {P_{i,F} = P\left( {i|{\mathbf{W}}_{L_1}, \cdots ,{\mathbf{W}}_{L_F}} \right) = \frac{{P\left( {{\mathbf{W}}_{L_1}, \cdots ,{\mathbf{W}}_{L_F}|i} \right)p\left( i \right)}}{{\mathop {\sum }\nolimits_{j = 1}^n \left[ {P\left( {{\mathbf{W}}_{L_1}, \cdots ,{\mathbf{W}}_{L_F}|j} \right)p\left( j \right)} \right]}}} \end{array}$$

Here *p*(*i*) = 1/*n* is the prior probability of the target being at location *i*. The vector ${\mathbf{W}}_{L_F} = \left( {W_{1,L_F}, \cdots ,W_{n,L_F}} \right)$ is the visual evidence at all locations gathered during the *F*^th^ fixation at location *L*_*F*_. Assuming the noise was independent at each location and during each fixation, we have:

16 $$\begin{array}{*{20}{c}} {P\left( {{\mathbf{W}}_{L_1}, \cdots ,{\mathbf{W}}_{L_F}|i} \right) = \mathop {\prod }\limits_{f = 1}^F P\left( {{\mathbf{W}}_{L_f}|i} \right) = \mathop {\prod }\limits_{f = 1}^F \mathop {\prod }\limits_{j = 1}^n P\left( {W_{j,L_f}|i} \right)} \end{array}$$

Since $W_{j,L_f}$ conditioned on target location *i* follows normal distribution $N\left( { \pm 0.5,1/d_{j,L_f}^{\prime 2}} \right)$, by substituting its probability density function into Eqs. (15) and (16) we could derive:

17 $$\begin{array}{*{20}{c}} {P_{i,F} = \frac{{p\left( i \right) \cdot \exp \left[ {\mathop {\sum }\nolimits_{f = 1}^F \left( {d_{i,L_f}^{\prime 2}W_{i,L_f}} \right)} \right]}}{{\mathop {\sum }\nolimits_{j = 1}^n \left\{ {p\left( j \right) \cdot \exp \left[ {\mathop {\sum }\nolimits_{f = 1}^F \left( {d_{j,L_f}^{\prime 2}W_{j,L_f}} \right)} \right]} \right\}}} = \frac{{P_{i,F - 1} \cdot \exp \left( {d_{i,L_F}^{\prime 2}W_{i,L_F}} \right)}}{{\mathop {\sum }\nolimits_{j = 1}^n \left[ {P_{j,F - 1} \cdot \exp \left( {d_{j,L_F}^{\prime 2}W_{j,L_F}} \right)} \right]}}} \end{array}$$

The ELM model selected the next fixation location to maximize the expected information gain of the next fixation. This could be calculated in a very simple form^1^:

18 $$L_{F + 1} = \mathop{{\mathrm{arg}}\, {\mathrm{max}}}\limits_{L_{F + 1}} \left\{ {H_{L_F}} - {\mathrm{E}}\left[ {H_{L_{F + 1}}} \right] \right\} = \mathop{{\mathrm{arg}}\, {\mathrm{max}}}\limits_{L_{F + 1}} \left\{ {\frac{1}{2}\mathop {\sum }_{i = 1}^{n} \left( {P_{i,T_F}} \cdot d_{i,L_{F + 1}}^{\prime 2} \right)} \right\}$$

where $H_{L_F} = - \mathop {\sum}\nolimits_{i = 1}^n {\left[ {P_{i,L_F} \cdot {\mathrm{ln}}(P_{i,L_F})} \right]}$ is the entropy of posterior probability of target location.

The model found the target whenever the posterior probability of the target being at the current fixation location exceeded a threshold *θ*_T_. The value of *θ*_T_ was chosen to make the model’s correct response rate comparable to the subjects’ data.

### The continuous-time ELM (CTELM) model

The CTELM model extends the ELM model to account for the distribution of fixation durations. A schematic diagram of the CTELM model is shown in Fig. 2 and will be described in the following sections.

#### Temporal course of evidence accumulation

The accumulation of visual evidence over time can be described by the evidence accumulation model discussed in previous sections. In practice, for each fixation *f*, we first calculated the values of parameter $a_{i,L_f}$ and $k_{i,L_f}$ at the 400 locations across the visual field according to Eqs. (11) and (12). These values were calculated only once per fixation. Then for each time step Δ*t* (1 ms), a random evidence sample ${\Delta}W_{i,L_f}$ was generated at each location *i* from the normal distribution $N\left( { \pm {\Delta}t,8{\Delta}t/a_{i,L_f}^2} \right)$ (see the relationship between *a* and *σ* in Eq. (10)) depending on whether target was present at this location. The current total accumulated evidence $W_{i,L_f,T_f}$ at location *i* at time *T*_*f*_ since the start of the *f*^th^ fixation was calculated from $W_{i,L_f,T_f - {\Delta}t}$ in the previous time step by:

19 $$\begin{array}{*{20}{c}} {W_{i,L_f,T_f} = {\Delta}W_{i,L_f} + W_{i,L_f,T_f - {\Delta}t} \cdot \exp \left( { - k_{i,L_f}{\Delta}t} \right)} \end{array}$$

#### Target’s posterior probability m*ap*

At each time step, the posterior probability of target being at the *i*^th^ (*i* = 1⋯*n*, *n* = 400) location could be calculated from the accumulated evidence from the current and all previous fixations (unlimited memory) by Bayesian theory:

20 $$\begin{array}{*{20}{c}} {P_{i,T_F} = \left( {i|{\mathbf{W}}_{L_1,T_1}, \cdots ,{\mathbf{W}}_{L_F,T_F}} \right) = \frac{{P\left( {{\mathbf{W}}_{L_1,T_1}, \cdots ,{\mathbf{W}}_{L_F,T_F}|i} \right)p\left( i \right)}}{{\mathop {\sum }\nolimits_{j = 1}^n \left[ {P\left( {{\mathbf{W}}_{L_1,T_1}, \cdots ,{\mathbf{W}}_{L_F,T_F}|j} \right)p\left( j \right)} \right]}}} \end{array},$$

where ${\mathbf{W}}_{L_F,T_F} = \left( {W_{1,L_F.T_F}, \cdots ,W_{n,L_F.T_F}} \right)$ is the accumulated evidence by time *T*_*F*_ of the *F*^th^ fixation at all locations. Assuming that visual information was independently accumulated at each location during each fixation, Eq. (20) could be simplified to (Supplementary Method):

21 $$\begin{array}{*{20}{c}} {P_{i,T_F} = \frac{{p\left( i \right) \cdot \exp \left[ {\mathop {\sum }\nolimits_{f = 1}^F \frac{{a_{i,L_f}^2W_{i,L_f,T_f}}}{{2 + 2\exp \left( { - T_f \cdot k_{i,L_f}} \right)}}} \right]}}{{\mathop {\sum }\nolimits_{j = 1}^n \left\{ {p\left( j \right) \cdot \exp \left[ {\mathop {\sum }\nolimits_{f = 1}^F \frac{{a_{j,L_f}^2W_{j,L_f,T_f}}}{{2 + 2\exp \left( { - T_f \cdot k_{j,L_f}} \right)}}} \right]} \right\}}}} \end{array}$$

By applying Eq. (21) to all target locations at each time step, we could calculate the change in the posterior probability map of the target location over time.

#### Decision on saccade timing

In the CTELM model, saccades were triggered randomly in two different ways according to their relative frequency. Ninety-seven percent of saccades (normal saccades) were triggered by a decision process concerning whether the current attention location contained the target. The first saccade in a trial was always a normal saccade. If the probability of the target being at the current attention location was lower than a threshold, the model makes a saccade decision. We defined the current attention location as the current fixation location when the next saccade decision had not been made, and as the next fixation location when the next saccade decision had been made but the eyes had not moved because of the delay from the cortex to the eye movement muscles (saccade lag). We implemented the saccade threshold *θ*_S_(*t*) as a “collapsing bound”^33,56–58^:

22 $$\begin{array}{*{20}{c}} {\log _{10}\left[ {\theta _{\mathrm{S}}\left( t \right)} \right] = q_1 \cdot \exp \left[ { - \left( {\frac{t}{{q_2}}} \right)^{q_3}} \right]} \end{array}$$

Here, *q*_1_, *q*_2_, *q*_3_ (*q*_1_, *q*_3_ < 0, *q*_2_ > 0) are parameters whose values are estimated by fitting to the fixation duration distribution in training set.

The remaining 3% saccades were low-latency saccades generated by other visual pathways^59,60^. This proportion was set according to the frequency of express saccades in an overlap task^61^. The latency (in seconds) is directly drawn from a gamma distribution with shape parameter 10 and scale parameter 6.9 × 10^−3^, plus a delay of 0.03 s. This latency follows a distribution with mean = 99 ms, 5% percentile = 68 ms, and 95% percentile = 138 ms, which roughly falls in the 80–120 ms range of express saccade latency^62^. When a saccade was triggered, a new saccade decision process started immediately before the delay of 0.03 s.

In the model, the delay from the retina to the visual cortex (eye-brain lag) was 0.06 s^63^, and the saccade lag was 0.03 s^64^. After the saccade decision was made, a new saccade decision process started immediately, and the model continued to accumulate information from the previous fixation until the arrival of new information after the eye-brain lag. We did not consider the saccade duration (a saccade is finished instantly) because of saccadic masking^11^.

#### Decision on saccade target

When making a saccade decision, the CTELM model used the ELM rule (Eq. (18)) to select the next fixation location. However, the deviation of Eq. (18) as shown by Najemnik and Geisler^1^ requires the visibility $d_{i,L_{f + 1}}^\prime$ to be a fixed value within a fixation, but in the CTELM model, $d_{i,L_{f + 1}}^\prime$ changes with time. As a simplification, we set the expected duration of the next fixation to be 0.25 s when evaluating Eq. (18). We ran Monte Carlo simulations and found that the expected information gain calculated in this way had a high Pearson correlation (mean = 0.92) with the actual information gain (Supplementary Method, Supplementary Fig. 18).

#### Decision on target location

The model found the target whenever the posterior probability of the target being at the current fixation location exceeded a threshold *θ*_T_. The value of *θ*_T_ was chosen to make the model’s correct response rate comparable to the subjects’ data.

### The constrained-CTELM (CCTELM) model

The schematic diagram of the CCTELM model is the same as the CTELM model (Fig. 2), but we limit the memory capacity, suppress the probability of long saccades, and add saccade inaccuracy to the model.

#### Memory capacity

For simplicity, the model either kept all information from a fixation or completely forgot it. A model with a memory capacity of M could only integrate information from the past *M* − 1 fixations plus the current fixation, so at the *F*^th^ fixation, the earliest fixation that the model could integrate was *f*_*s*_ = max(1, *F* − *M* + 1). The posterior probability of target being at location *i* could be calculated as:

23 $$\begin{array}{*{20}{c}} {P_{i,T_F} = \left( {i|{\mathbf{W}}_{L_{f_s},T_{f_s}}, \cdots ,{\mathbf{W}}_{L_F,T_F}} \right) = \frac{{P\left( {{\mathbf{W}}_{L_{f_s},T_{f_s}}, \cdots ,{\mathbf{W}}_{L_F,T_F}|i} \right)p\left( i \right)}}{{\mathop {\sum }\nolimits_{j = 1}^n \left[ {P\left( {{\mathbf{W}}_{L_{f_s},T_{f_s}}, \cdots ,{\mathbf{W}}_{L_F,T_F}|j} \right)p\left( j \right)} \right]}}} \end{array}$$

This expression could be simplified to (Supplementary Method):

24 $$\begin{array}{*{20}{c}} {P_{i,T_F} = \frac{{p\left( i \right) \cdot \exp \left[ {\mathop {\sum }\nolimits_{f = f_s}^F \frac{{a_{i,L_f}^2W_{i,L_f,T_f}}}{{2 + 2\exp \left( { - T_f \cdot k_{i,L_f}} \right)}}} \right]}}{{\mathop {\sum }\nolimits_{j = 1}^n \left\{ {p\left( j \right) \cdot \exp \left[ {\mathop {\sum }\nolimits_{f = f_s}^F \frac{{a_{j,L_f}^2W_{j,L_f,T_f}}}{{2 + 2\exp \left( { - T_f \cdot k_{j,L_f}} \right)}}} \right]} \right\}}}} \end{array}$$

#### Decision on saccade timing

The CCTELM model used the same method to decide saccade timing as the CTELM model. However, because of the saccadic inaccuracy, the CCTELM model could fixate at every possible location in the search field (not just the predefined 400 locations). The posterior probability of target being at current attention location, therefore, was the sum of posterior probability at all the predefined 400 locations within 0.5 degrees away from the actual attention location. Our Monte Carlo simulation showed that in 44.46%, 47.13% and 8.41% cases, one, two, and three nearby locations were counted, respectively.

#### Decision on saccade target

We applied a penalty function H(*L*_*f*_, *L*_*f*+1_) as a general representation of possible saccadic cost to the ELM rule when selecting the next fixation location:

25 $$L_{f + 1} = \mathop{{\mathrm{arg}}\,{\mathrm{max}}}\limits_{L_{f + 1}} \left\{ {{\mathrm{H}}\left( {L_f,L_{f + 1}} \right) \cdot \frac{1}{2}\mathop {\sum }\limits_{i = 1}^n \left( {P_{i,T_f} \cdot d_{i,L_{f + 1}}^{\prime 2}} \right)} \right\}$$

26 $${{\mathrm{H}}\left( {L_f,L_{f + 1}} \right) = \exp \left( { - \frac{{\max \left[ {{\mathrm{D}}\left( {L_f,L_{f + 1}} \right),1} \right]}}{c}} \right)},$$

where D(*L*_*f*_, *L*_*f*+1_) is the angular distance between the current and next potential fixation location, and *c* is a free parameter. We did not apply the penalty function within 1° of the fixation location to prevent interference with the inhibition of return phenomenon generated by the model. The exponential function was chosen because: (1) The saccade amplitude distribution was very similar to the exponential distribution. (2) We also tested linear and reciprocal functions as the penalty function, but their results were inferior to those of the exponential function (the goodness-of-fit of saccade amplitude distribution: exponential penalty: *B*_c_ = 0.9930, reciprocal penalty: *B*_c_ = 0.9858, linear penalty: *B*_c_ = 0.9918; Supplementary Fig. 19).

#### Saccade inaccuracy

The accuracy of saccades can be described by the bias between the mean saccade landing positions and the saccade target and the SD of the saccade landing positions. We considered the bias and SD of the saccade landing position in the CCTELM model, so it could fixate at any location inside the search image instead of the 400 predefined locations. We further assumed that the actual saccade landing positions formed a 2-dimensional Gaussian distribution around the mean^19^ and ignored any difference in the horizontal and vertical directions.

To accurately describe the relationship between bias and SD of saccade landing positions and saccade target eccentricity, we analyzed the raw data from a previous study^19^. In brief, subjects were required to saccade at a target with eccentricity varying from 0.5° to 17.5° left or right away from the initial fixation location, and identified the number of small dots presented around the target location^19^. Only the first, non-anticipatory saccades after presentation of the target were analyzed. Raw data was shown in Supplementary Fig. 6a. Leftward and rightward trials with the same eccentricity were combined (leftward saccades flipped to rightward). The bias of saccade landing position was calculated as the mean of horizontal saccade amplitude minus the saccade target eccentricity. The SD of saccade landing positions *σ*_s_ was calculated as:

27 $$\begin{array}{*{20}{c}} {\sigma _{\mathrm{s}} = \sqrt {\frac{{\mathop {\sum }\nolimits_{i = 1}^N \left[ {\left( {x_i - \bar x} \right)^2 + \left( {y_i - \bar y} \right)^2} \right]}}{N}} } \end{array}$$

Here (*x*_*i*_*, y*_*i*_) is the saccade landing position of the *i*^th^ trial, $(\bar x,\bar y)$ is the mean saccade landing position of all *N* trials with the same target eccentricity. By linear regression, the relationship between target angular eccentricity *x* and *σ*_s_ could be described by (Supplementary Fig. 6b):

28 $$\begin{array}{*{20}{c}} {\sigma _{\mathrm{s}} = 0.0453x + 0.364} \end{array}$$

And the relationship between target angular eccentricity *x* and saccade landing could be described by (Supplementary Fig. 6c):

29 $${\mathrm{Bias}} = - \max \left( {0,x - 10} \right) \times 0.1195$$

#### Decision on target location

The CCTELM model stopped searching whenever the posterior probability at the current fixation location (sum of all 400 predefined locations within 0.5 degrees from fixation location) exceeded a target detection threshold *θ*_T_. The model set the target location as the predefined location with highest posterior probability. The value of *θ*_T_ was chosen to make the model’s correct response rate comparable to the subjects’ data.

### Statistics and reproducibility

#### Fitting model parameters

Supplementary Table 1 shows a summary of the parameters used in the ELM, CTELM, and CCTLEM model. All the parameters were derived by fitting the models to the eye movement data of training set (including correct and error trials).

For the CTELM model, there are three free parameters in Eq. (22) that control the distribution of the fixation duration. The remaining parameters were estimated from experimental data and fixed when fitting the free parameters. The values of the free parameters were estimated by genetic algorithm^65^ that minimized the sum of the Bhattacharyya distance (*B*_d_)^32^ between the simulated and experimental histograms of the fixation duration (*T*_sim_, *T*_exp_):

30 $$\begin{array}{*{20}{c}} {{\mathrm{Loss}} = B_{\mathrm{d}}\left( {T_{{\mathrm{sim}}},T_{{\mathrm{exp}}}} \right)} \end{array}$$

where the *B*_d_ of the two histograms *H*_1_ and *H*_2_ (with *N* bins) is defined as:

31 $$\begin{array}{*{20}{c}} {B_{\mathrm{d}}\left( {H_1,H_2} \right) = - \ln \left[ {B_{\mathrm{c}}\left( {H_1,H_2} \right)} \right]} \end{array}$$

32 $$\begin{array}{*{20}{c}} {B_{\mathrm{c}}\left( {H_1,H_2} \right) = \mathop {\sum }\limits_{i = 1}^N \sqrt {H_1\left( i \right) \cdot H_2\left( i \right)} } \end{array}$$

The histogram should be calculated as probability. Intuitively, the Bhattacharyya coefficient quantifies the degree of overlap between two histograms. If the two histograms are identical, then *B*_c_ = 1, and if there is no overlap between the two histograms, then *B*_c_ = 0. The genetic algorithm was implemented in the Global Optimization Toolbox in MATLAB. We set the population size to 150 and the maximum number of generations to 25, leaving the other parameters unchanged. For each parameter sample in the population, the model was run 1000 times to obtain the histogram of fixation durations. We used the Multicore package (https://www.mathworks.com/matlabcentral/fileexchange/13775-multicore-parallel-processing-on-multiple-cores) to accelerate the calculation. Fitting the parameters took ~2.5 h when running on 11 machines.

For the CCTELM model, we fit the four free parameters in Eqs. (22) and (26) by genetic algorithm. The cost function is the sum of the *B*_d_ between the simulated and experimental histograms of the fixation duration (*T*_sim_, *T*_exp_) and saccade amplitude (*A*_sim_, *A*_exp_):

33 $$\begin{array}{*{20}{c}} {{\mathrm{Loss}} = B_{\mathrm{d}}\left( {T_{{\mathrm{sim}}},T_{{\mathrm{exp}}}} \right) + B_{\mathrm{d}}\left( {A_{{\mathrm{sim}}},A_{{\mathrm{exp}}}} \right)} \end{array}$$

We set the population size to 200 and max generations to 25, leaving the other parameters unchanged. For each parameter sample in the population, the model was run 1000 times to obtain the histograms of the fixation durations and saccade amplitudes.

To find the memory capacity for the CCTELM model, we first assumed unlimited memory capacity and fit the four free parameters. We then evaluated the model with memory capacity of 2, 4, ⋯, 14 and infinite fixations for 50000 times each. The value of target detection threshold *θ*_T_ was set to 0.952 (Supplementary Table 1). For the simulation result of each capacity, we calculated the histograms of fixation duration, fixation distance and saccade amplitude of the initial 20 fixations and saccades, and the overall distribution of fixation location. All trials were used. Then for fixation duration/distance and saccade amplitude, we calculated the *B*_c_ between histograms of simulation and experiment data at each ordinal position. Thus, for each memory capacity value, we obtained one *B*_c_ for fixation location and 20 *B*_c_ for each of fixation duration, fixation distance, and saccade amplitude. Finally, for each of the above three eye movement metrics, the 20 *B*_c_ were averaged to obtain a single *B*_c_.

#### Simulating visual search

The three models were evaluated for 100,000 trials each. We randomly rotate the 400 predefined locations around the image center before each trial to help obtain a smooth distribution of fixation locations. The target locations were randomly selected with equal probability. Eye movement metrics were summarized by the same method used in experiment data. We used *B*_c_ to quantify the similarity between the histograms of the model’s and humans’ eye movement metrics.

#### Model comparison

To compare the relative importance of the three constraints in the CCTELM model, we evaluated the CCTELM model without each of the constraints 100,000 times (using the same parameter values as the full CCTELM model), and calculated the average *B*_c_ of fixation duration, saccade amplitude, fixation location and fixation distance (to the center of the image) distribution. To estimate the confidence interval of *B*_c_, we bootstrapped both the experimental and simulation data 2000 times and calculated 2000 *B*_c_ values. The 99% confidence interval was calculated by 2.576 × SD of the *B*_c_ values.

## Supplementary information

Supplementary Information

## Data Availability

The experimental and simulation results are available on Open Science Framework^66^ at https://osf.io/ypcwx/.

## Code availability

The code for the visual search models is available on Open Science Framework at https://osf.io/ypcwx/.
