# Supplementary material for: Human visual search follows a suboptimal Bayesian strategy revealed by a spatiotemporal computational model and experiment
Source: Commun Biol. 2021 Jan 4;4:34. doi: 10.1038/s42003-020-01485-0 (PMC7782508; doi:10.1038/s42003-020-01485-0)
Supplement: Supplementary file 1 — Supplementary Information [file 42003_2020_1485_MOESM1_ESM.pdf]

Supplementary Method

Calculation of the Posterior Probability Map

Here we show the details of derivation of the expression to calculate posterior probability map of target location in CTELM and CCTELM models (equations (21) and (24) in the main text).

CTELM Model

According to equation (20) in the main text, at time T_F after the start of the Fth fixation, the posterior probability of target being at location i given accumulated information **W** from all previous fixations is:

$$P_{i,T_F} = P\left(i \mid \mathbf{W}_{L_1,T_1},\cdots,\mathbf{W}_{L_F,T_F}\right) = \frac{P\left(\mathbf{W}_{L_1,T_1},\cdots,\mathbf{W}_{L_F,T_F} \mid i\right)p(i)}{\sum_{j=1}^{n}\left[P\left(\mathbf{W}_{L_1,T_1},\cdots,\mathbf{W}_{L_F,T_F} \mid j\right)p(j)\right]} \tag{A1}$$

We assume that visual information is independently accumulated at each location during each fixation, so the joint probability in equation (A1) can be simplified to:

$$P_{i,T_F} = P\left(i \mid \mathbf{W}_{L_1,T_1},\cdots,\mathbf{W}_{L_F,T_F}\right) = \frac{\prod_{f=1}^{F}\prod_{q=1}^{n}P\left(W_{q,L_f,T_f} \mid i\right)p(i)}{\sum_{j=1}^{n}\left[\prod_{f=1}^{F}\prod_{q=1}^{n}P\left(W_{q,L_f,T_f} \mid j\right)p(j)\right]} \tag{A2}$$

From equations (7)-(9) in the main text we know the accumulated information W_{q,L_f,T_f} at location q when the fth fixation is at L_f follows the normal distribution:

$$W_{q,L_f,T_f} \sim N\left(\lambda_{q|i}\frac{1-\exp\left(-k_{q,L_f}T_f\right)}{k_{q,L_f}},\frac{4\left[1-\exp\left(-2k_{q,L_f}T_f\right)\right]}{a_{q,L_f}^2 k_{q,L_f}}\right) \tag{A3}$$

where $\lambda_{q|i} = 1$ if $q = i$, otherwise $\lambda_{q|i} = -1$, $T_f = n_t \cdot \Delta t$, and $a_{q,L_f} = 4/\sqrt{2}\sigma_{q,L_f}$. Here the parameters a and k have subscripts q and L_f because their values depend on the relative location between fixation location L_f and the queried location q (equations (11), (12) in the main text). Note that W_{q,L_F,T_F} only contains the accumulated information from the start of the Fth fixation. Information from all previous fixations are stored in W_{q,L_f,T_f} where $f = 1\cdots F-1$, and they are all used to calculate the posterior probability during the Fth fixation in equation (A2).

Therefore, we can write the probability density function of W_{q,L_f,T_f}, denoted by $P\left(W_{q,L_f,T_f} \mid i\right)$, as:

$$P\left(W_{q,L_f,T_f} \mid i\right) = \frac{1}{\sqrt{2\pi \frac{4\left[1-\exp\left(-2k_{q,L_f}T_f\right)\right]}{a_{q,L_f}^2 k_{q,L_f}}}} \cdot \exp\left\{\frac{\left[W_{q,L_f,T_f} - \lambda_{q|i}\frac{1-\exp\left(-k_{q,L_f}T_f\right)}{k_{q,L_f}}\right]^2}{2\cdot\frac{4\left[1-\exp\left(-2k_{q,L_f}T_f\right)\right]}{a_{q,L_f}^2 k_{q,L_f}}}\right\}$$

$$= \frac{a_{q,L_f}}{2}\sqrt{\frac{k_{q,L_f}}{2\pi\left[1-\exp\left(-2k_{q,L_f}T_f\right)\right]}} \cdot \exp\left\{\frac{a_{q,L_f}^2 k_{q,L_f}\left[W_{q,L_f,T_f} - \lambda_{q|i}\frac{1-\exp\left(-k_{q,L_f}T_f\right)}{k_{q,L_f}}\right]^2}{8\left[1-\exp\left(-2k_{q,L_f}T_f\right)\right]}\right\} \quad \text{(A4)}$$

$$= \frac{a_{q,L_f}}{2}\sqrt{\frac{k_{q,L_f}}{2\pi\left(1-A_{q,L_f,T_f}^2\right)}} \cdot \exp\left[\frac{a_{q,L_f}^2 k_{q,L_f}\left(W_{q,L_f,T_f} - \lambda_{q|i}\frac{1-A_{q,L_f,T_f}}{k_{q,L_f}}\right)^2}{8\left(1-A_{q,L_f,T_f}^2\right)}\right]$$

Note in the last step of equation (A4) we define $A_{q,L_f,T_f} = \exp\left(-k_{q,L_f}T_f\right)$.

Substituting equation (A4) into equation (A2):

$$P_{i,T_F} = \frac{p(i)\left\{\prod_{f=1}^{F}\prod_{q=1}^{n}\left[\frac{a_{q,L_f}}{2}\sqrt{\frac{k_{q,L_f}}{2\pi\left(1-A_{q,L_f,T_f}^2\right)}}\right]\right\}\cdot\exp\left\{-\sum_{f=1}^{F}\sum_{q=1}^{n}\left[\frac{a_{q,L_f}^2 k_{q,L_f}\left(W_{q,L_f,T_f}-\lambda_{q|i}\frac{1-A_{q,L_f,T_f}}{k_{q,L_f}}\right)^2}{8\left(1-A_{q,L_f,T_f}^2\right)}\right]\right\}}{\sum_{j=1}^{n}\left\{p(j)\left\{\prod_{f=1}^{F}\prod_{q=1}^{n}\left[\frac{a_{q,L_f}}{2}\sqrt{\frac{k_{q,L_f}}{2\pi\left(1-A_{q,L_f,T_f}^2\right)}}\right]\right\}\cdot\exp\left\{-\sum_{f=1}^{F}\sum_{q=1}^{n}\left[\frac{a_{q,L_f}^2 k_{q,L_f}\left(W_{q,L_f,T_f}-\lambda_{q|j}\frac{1-A_{q,L_f,T_f}}{k_{q,L_f}}\right)^2}{8\left(1-A_{q,L_f,T_f}^2\right)}\right]\right\}\right\}}$$

$$= \frac{p(i)\cdot\exp\left\{-\sum_{f=1}^{F}\sum_{q=1}^{n}\left[\frac{a_{q,L_f}^2 k_{q,L_f}\left(W_{q,L_f,T_f}-\lambda_{q|i}\frac{1-A_{q,L_f,T_f}}{k_{q,L_f}}\right)^2}{8\left(1-A_{q,L_f,T_f}^2\right)}\right]\right\}}{\sum_{j=1}^{n}\left\{p(j)\cdot\exp\left\{-\sum_{f=1}^{F}\sum_{q=1}^{n}\left[\frac{a_{q,L_f}^2 k_{q,L_f}\left(W_{q,L_f,T_f}-\lambda_{q|j}\frac{1-A_{q,L_f,T_f}}{k_{q,L_f}}\right)^2}{8\left(1-A_{q,L_f,T_f}^2\right)}\right]\right\}\right\}} \quad \text{(A5)}$$

$$= \frac{1}{1+\sum_{j=1,j\neq i}^{n}\left\{\frac{p(j)}{p(i)}\exp\left\{\sum_{f=1}^{F}\sum_{q=1}^{n}\left\{\frac{a_{q,L_f}^2 k_{q,L_f}}{8\left(1-A_{q,L_f,T_f}^2\right)}\left[\left(W_{q,L_f,T_f}-\lambda_{q|i}\frac{1-A_{q,L_f,T_f}}{k_{q,L_f}}\right)^2-\left(W_{q,L_f,T_f}-\lambda_{q|j}\frac{1-A_{q,L_f,T_f}}{k_{q,L_f}}\right)^2\right]\right\}\right\}\right\}}$$

Note that:

$$\left(W_{q,L_f,T_f}-\lambda_{q|i}\frac{1-A_{q,L_f,T_f}}{k_{q,L_f}}\right)^2-\left(W_{q,L_f,T_f}-\lambda_{q|j}\frac{1-A_{q,L_f,T_f}}{k_{q,L_f}}\right)^2 = 2\left(\lambda_{q|j}-\lambda_{q|i}\right)W_{q,L_f,T_f}\frac{1-A_{q,L_f,T_f}}{k_{q,L_f}} \quad \text{(A6)}$$

Substituting equation (A6) into equation (A5), and note that ($\lambda_{q|i}$ - $\lambda_{q|j}$) = 0 for $q \neq i$ and $q \neq j$, we have:

$$
\begin{aligned}
P_{i,T_F} &= \frac{1}{1+\sum_{j=1,j\neq i}^{n}\left\{\frac{p(j)}{p(i)}\exp\left\{\sum_{f=1}^{F}\sum_{q=1}^{n}\left[\frac{a_{q,L_f}^{2}\left(\lambda_{q|j}-\lambda_{q|i}\right)W_{q,L_f,T_f}}{4\left(1+A_{q,L_f,T_f}\right)}\right]\right\}\right\}} \\
&= \frac{1}{1+\sum_{j=1,j\neq i}^{n}\left\{\frac{p(j)}{p(i)}\exp\left\{\sum_{f=1}^{F}\left[\frac{a_{j,L_f}^{2}W_{j,L_f,T_f}}{2\left(1+A_{j,L_f,T_f}\right)}-\frac{a_{i,L_f}^{2}W_{i,L_f,T_f}}{2\left(1+A_{i,L_f,T_f}\right)}\right]\right\}\right\}} \\
&= \frac{p(i)\cdot\exp\left[\sum_{f=1}^{F}\frac{a_{i,L_f}^{2}W_{i,L_f,T_f}}{2\left(1+A_{i,L_f,T_f}\right)}\right]}{\sum_{j=1}^{n}\left\{p(j)\cdot\exp\left[\sum_{f=1}^{F}\frac{a_{j,L_f}^{2}W_{j,L_f,T_f}}{2\left(1+A_{j,L_f,T_f}\right)}\right]\right\}} \\
&= \frac{p(i)\cdot\exp\left[\sum_{f=1}^{F}\frac{a_{i,L_f}^{2}W_{i,L_f,T_f}}{2+2\exp\left(-k_{i,L_f}T_f\right)}\right]}{\sum_{j=1}^{n}\left\{p(j)\cdot\exp\left[\sum_{f=1}^{F}\frac{a_{j,L_f}^{2}W_{j,L_f,T_f}}{2+2\exp\left(-k_{j,L_f}T_f\right)}\right]\right\}}
\end{aligned}
\tag{A7}
$$

and this is the formula to calculate posterior probability map in equation (21) in the main text.

CCTELM Model

The CCTELM model with a memory capacity of M can only integration information from current fixation plus $M-1$ previous fixations, so the earliest fixation that the model could integrate is $f_s = \max(1, F-M+1)$. Therefore, equation (A2) should be written as:

$$
P_{i,T_F} = P\left(i \mid \mathbf{W}_{L_{f_s},T_{f_s}},\cdots,\mathbf{W}_{L_F,T_F}\right) = \frac{\prod_{f=f_s}^{F}\prod_{q=1}^{n}P\left(W_{q,L_f,T_f} \mid i\right)p(i)}{\sum_{j=1}^{n}\left[\prod_{f=f_s}^{F}\prod_{q=1}^{n}P\left(W_{q,L_f,T_f} \mid j\right)p(j)\right]} \tag{A8}
$$

With the same process from equation (A2) to equation (A7), the posterior probability of target being at location i at time T_F after the start of the Fth fixation is:

$$
P_{i,T_F} == \frac{p(i)\cdot\exp\left[\sum_{f=f_s}^{F}\frac{a_{i,L_f}^{2}W_{i,L_f,T_f}}{2+2\exp\left(-k_{i,L_f}T_f\right)}\right]}{\sum_{j=1}^{n}\left\{p(j)\cdot\exp\left[\sum_{f=f_s}^{F}\frac{a_{j,L_f}^{2}W_{j,L_f,T_f}}{2+2\exp\left(-k_{j,L_f}T_f\right)}\right]\right\}} \tag{A9}
$$

And this is equation (24) in the main text to calculate posterior probability map for a model with limited memory.

Validation of the ELM Rule

The calculation of expected information gain of the next fixation in equation (18) in the main text was derived by Najemnik & Geisler (2009) [2] without the consideration of fixation duration, so the target visibility map was treated as fixed values. However, in our model the target visibility changes according to fixation duration, which is a random variable, so mathematically the derivation no longer holds. Here we used a Monte Carlo simulation to show that equation (18) still has a high level of accuracy even when the fixation duration varies stochastically.

The Monte Carlo simulation contained 100 sessions. In each session, we generated a random prior probability map of target location and calculated the actual and expected information gain of across all possible fixation locations. At each fixation location, the expected information gain was calculated by equation (18), and the actual information gain was obtained by repeatedly simulating the fixation for more than 2000 trials until the average information gain changed less than 0.1%. Within each repetition, the target location was randomly chosen according to the prior probability map. We used the same visibility map, saccade threshold parameters, and fixation termination rule as in the CTELM model (Supplementary Table 1).

At the end of each session we could get a map of expected information gain and a map of actual information gain. We then calculated the Pearson correlation coefficient of these two maps. Supplementary Fig 18 shows the correlation between expected and actual information gain from the results of randomly selected 9 sessions. The mean correlation coefficient across 100 sessions of simulation is 0.917, with a maximum of 0.950 and a minimum of 0.847.

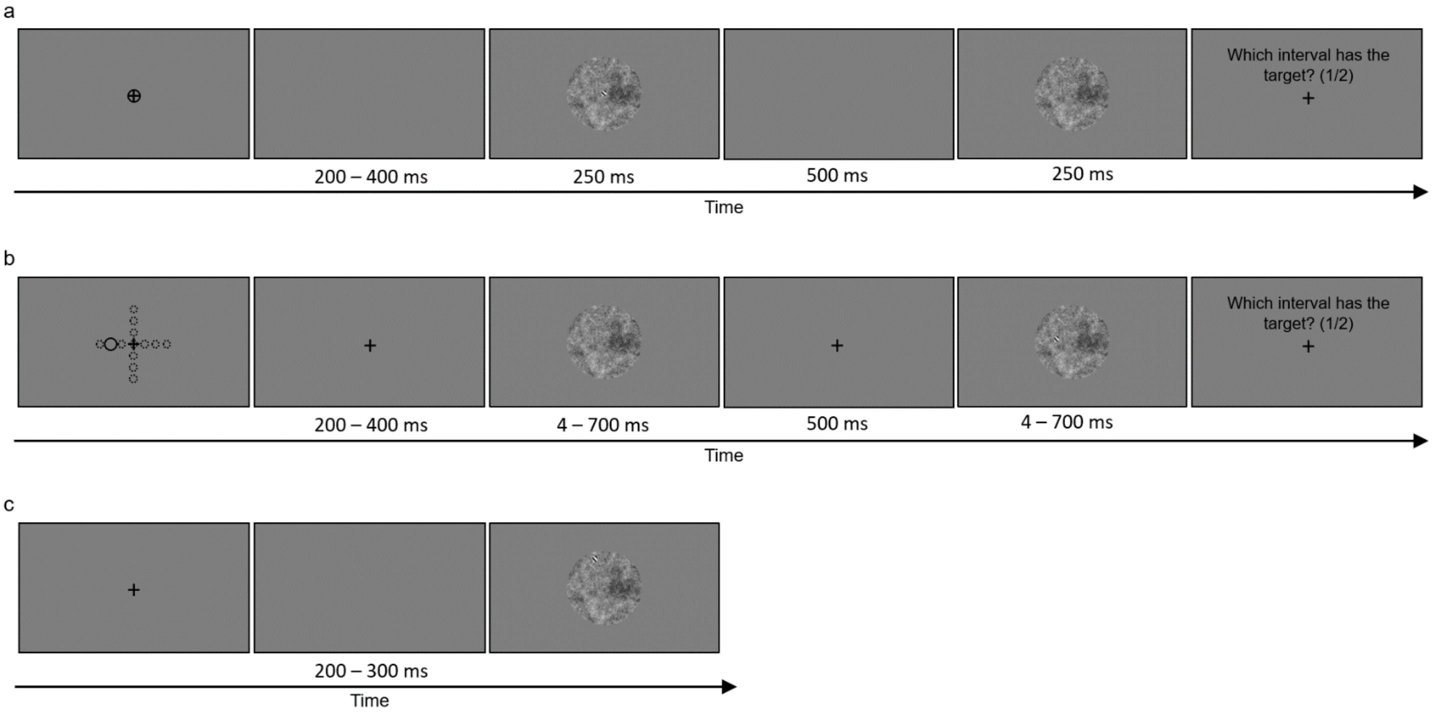

Supplementary Fig 1. Visibility map and visual search experiment trial procedure. **a**. Selecting target root-mean-squared (RMS) contrast. Subjects had to keep fixating at the center of the image throughout the trial and observe two images displayed for 250 ms (separated by a blank screen of 500 ms). The two images had the same background noise but a randomly chosen one had a target grating at the center. The target RMS contrast ranged from 0.03 to 0.12. Subjects then chose the image (1st or 2nd) that had the target. A trial was aborted if the subject fixated more than 1° away from the screen center at any time during the trial. After this experiment, we chose a target RMS contrast level for each subject that gave foveal target visibility = 3.0 (see methods). **b**. Detection task procedure. This task had three versions, and the first version which was used in this manuscript was shown. The overall procedure was the same as in panel **a**, but at the start of each trial, a black circle cue indicates where the target will occur in the following two images. The target RMS contrast is fixed for each subject, but the visible time of the two images will vary from 4 to 700 ms (same within a trial, vary across trials). The cue location will be chosen from a set of predefined horizontal and vertical midline locations (including image center) of the image (the smaller dashed black circle, not shown in actual experiment). Subject had to fixate at image center throughout the trial. To facilitate fixation, a cross would appear at the image center during the blank intervals if the target location was at peripheral location. The cross would not show if target appeared at the image center to prevent visual masking. The second version was the same as the first version except that the target location cue was not shown at the start of each trial, and the fixation cross was not shown at during the two blank intervals. The third version was the same as the first version except that the image visible time was fixed to 250 ms in each trial. **c**. Visual search

experiment. The target grating would appear at a uniformly random location within the search image and the subject needed to find it, fixate at it and press a key to respond. No limit was imposed on searching time. In **a**, **b** and **c,** background noise image used in each trial was randomly chosen from a pre-generated dataset of 1000 images.

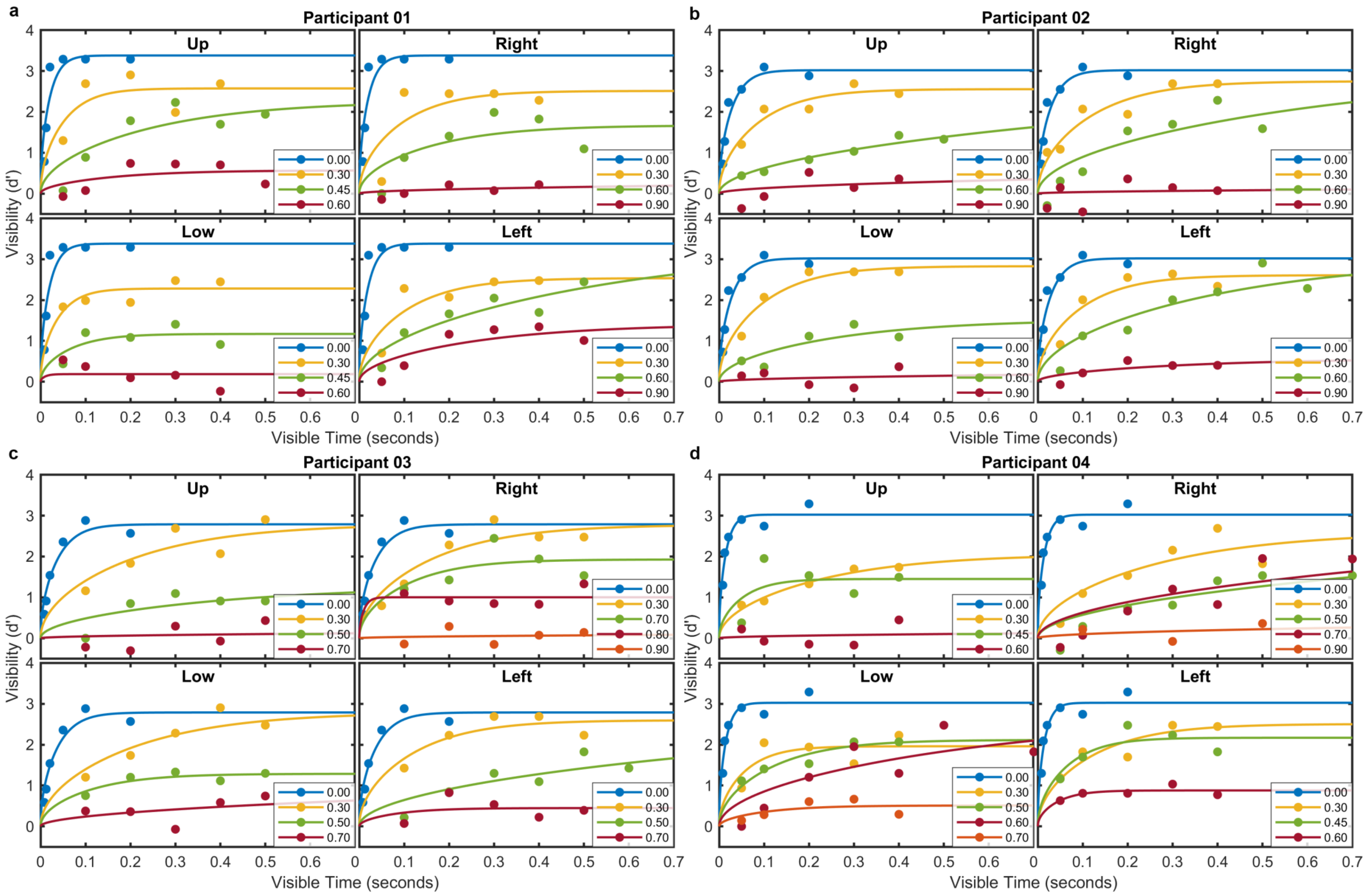

Supplementary Fig 2. Temporal course of target visibility at four cardinal directions relative to fixation location of subject 1-4 (in panels a-d) in training set. Each color (gray scale) represents data measured at different eccentricities (shown in legend in each subplot). The numbers in legend represent the relative distance between measured location to fixation location in unit of the image radius (0.00 means fixation location). The data measured at fixation location are shown in every subplot. Dots represent raw data and lines are equation (10) fitted to the raw data measured at each location.

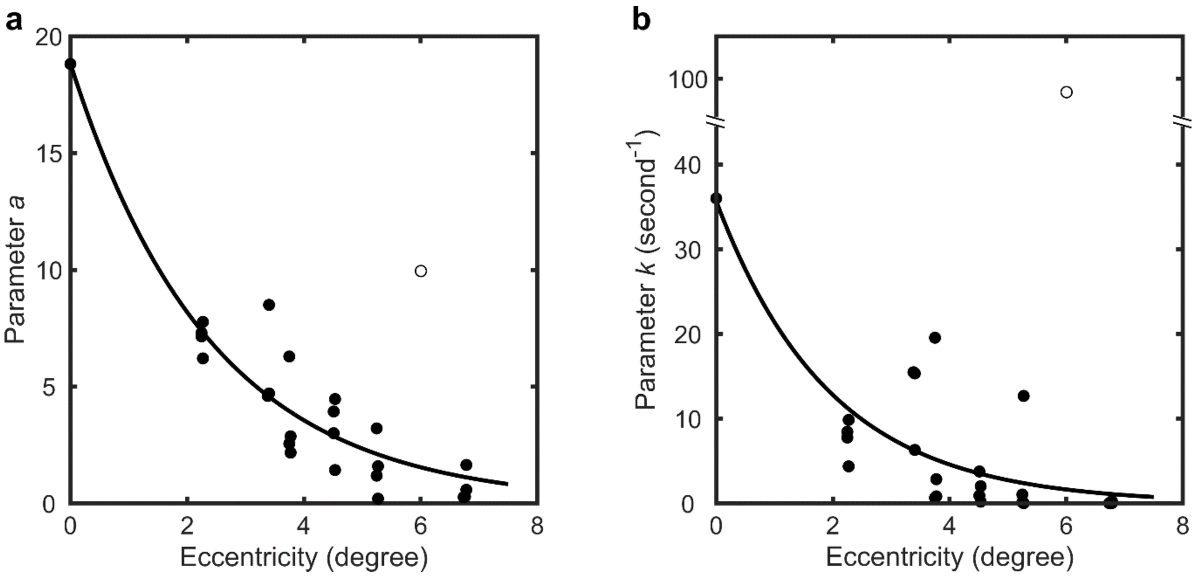

Supplementary Fig 3. Relationship between the values of parameters *a* (panel **a**) and *k* (panel **b**) in equation (10) in the main text to target's distance from fixation location. Each dot in the plot represents the values of parameters *a* and *k* obtained by separately fitting equation (10) to the time course of target visibility measured at each location. The open circles represent outliers. The curve is an exponential function $y = \beta_1 \cdot \exp(-\beta_2 \cdot x)$ fit to the dots (β_1 and β_2 are parameters, the outliers are ignored). Here we grouped target location only by their distance to fixation center and ignored the direction.

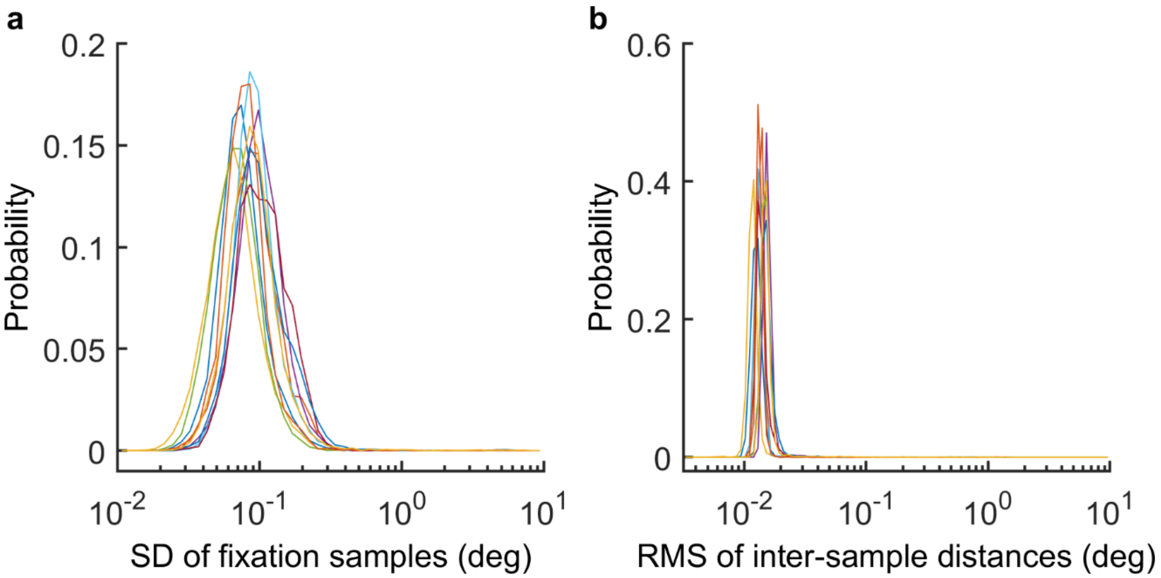

Supplementary Fig 4. Eye tracking data quality of all the 10 subjects. **a**. Distribution of standard deviation (SD) of all fixation samples of each subject. **b**. Distribution of root-mean-squared (RMS) of inter-sample angular distances of all fixation samples of each subject. In panel **a** and **b** each line represents one subject. The value of SD and RMS were first calculated for each eye and then average between the two eyes.

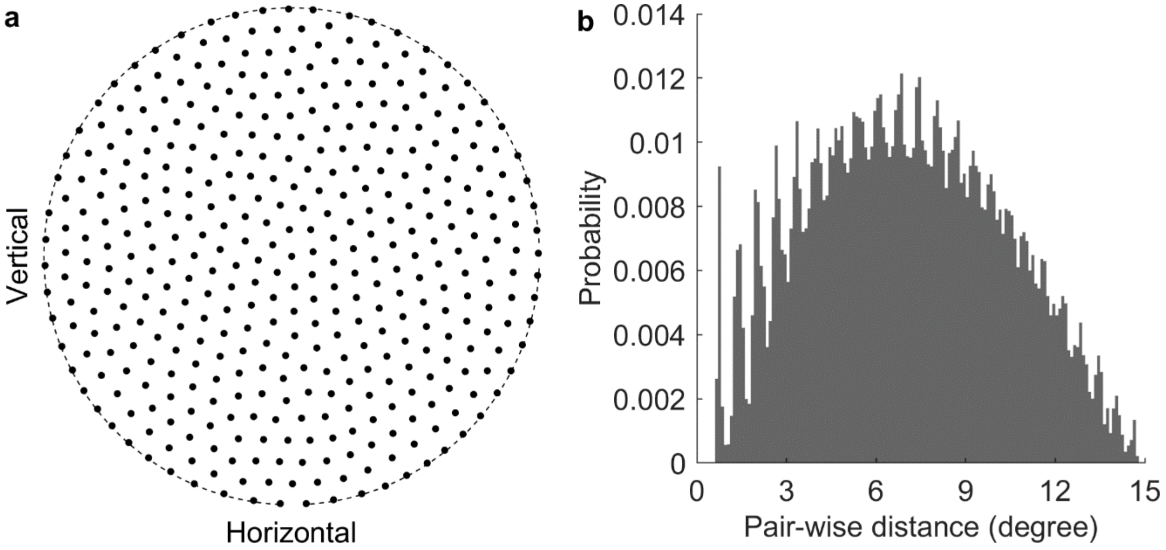

Supplementary Fig 5. Predefined locations used in the models. **a**. Black dots are the 400 positions where the ELM and CTELM model can fixate, and where target location is chosen in the ELM, CTELM, and CCTELM models. The dashed circle is the boundary of the search area. We generated these positions by simulating 400 dots repelling each other. The repelling force was proportional to the inverse sixth power of the distance between two dots. One dot is fixed at the image center and served as the starting position of the visual search. **b**. Distribution of pair-wise angular distance between all predefined locations in panel **a**.

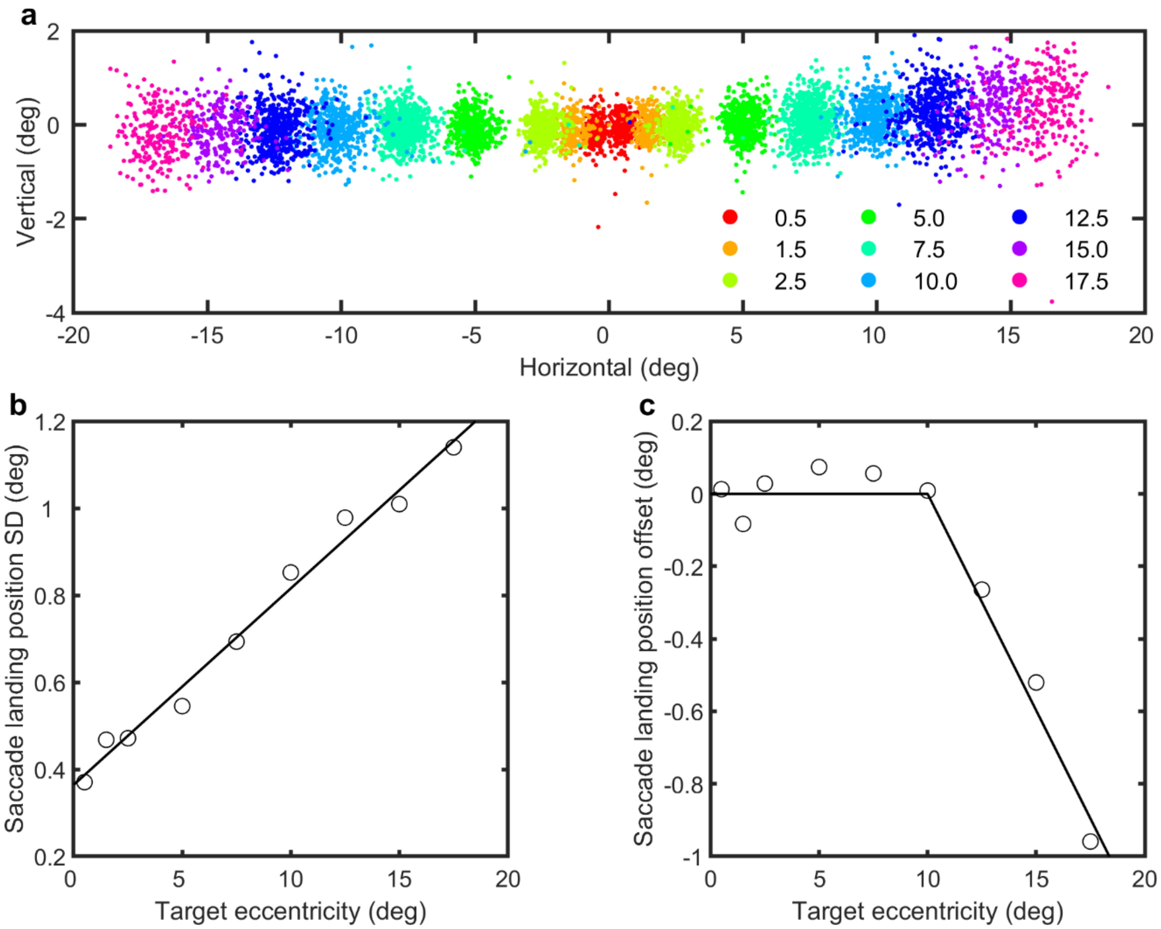

Supplementary Fig 6. Variability of saccadic landing position as a function of saccade target eccentricity. The plots are generated by re-analyzing data from [1]. **a**. Raw saccade landing position to target at 0.5° – 17.5° (shown in legend) leftward and rightward from initial fixation location (0, 0). Each dot represents data from one trial. **b.** Standard deviation (SD) of saccade landing position as a function of target eccentricity. Leftward and rightward of the same target eccentricity are combined. The SD of saccade landing position was calculated by equation (27) in the main text. Circles are experiment data, and the line is the best-fitting line. **c**. Average offset of saccade landing position relative to target eccentricity. Negative values mean that the saccade undershoot the target. Leftward and rightward of the same target eccentricity are combined. Circles are experiment data, and the line is a truncated linear function fitted to the data (equation (29) in the main text).

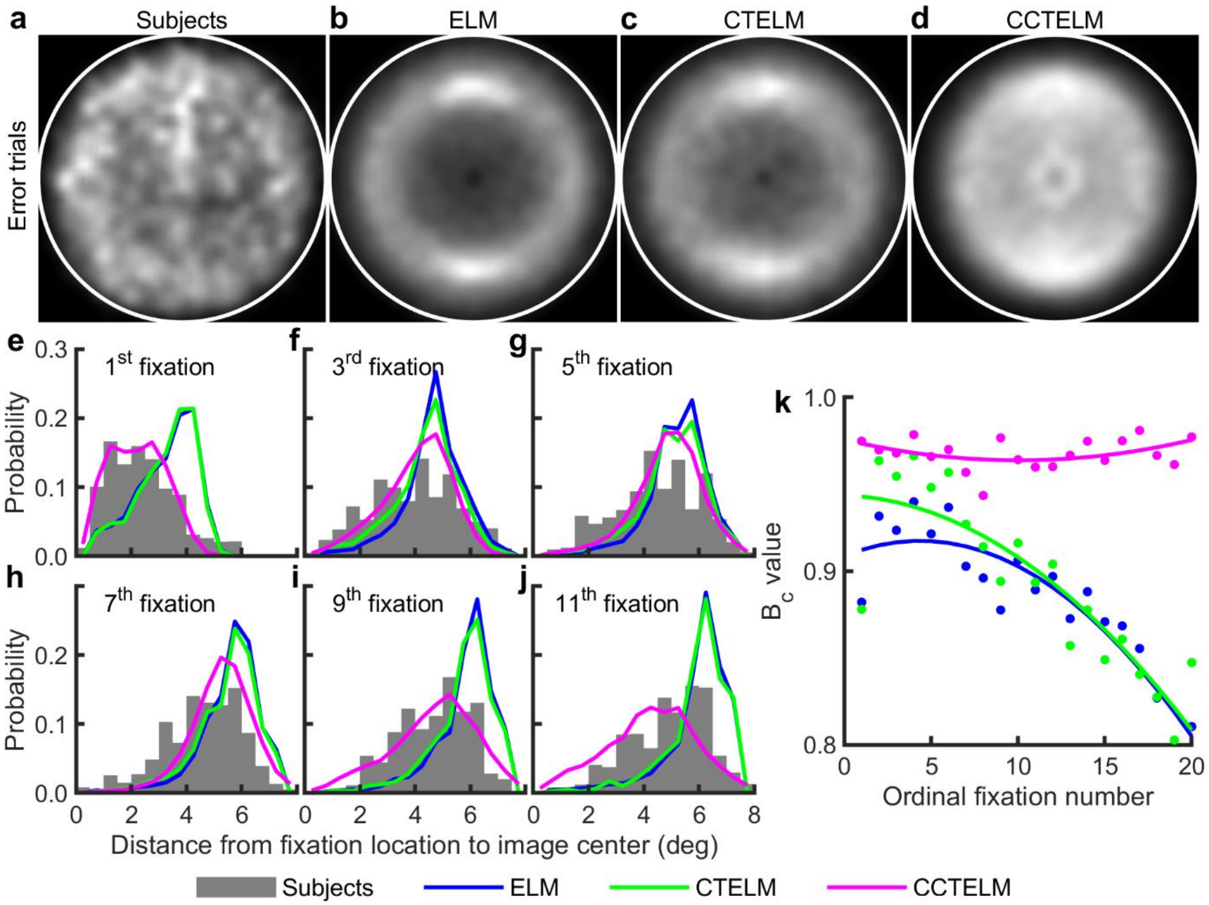

Supplementary Fig 7. Fixation location distribution of subjects and models in error trials. **a-d:** Distribution of fixations location in the search field (inside the white circle), lighter means higher density. The densities were obtained by smoothing the scatterplot of the fixation locations by a Gaussian window with a standard deviation of 0.35° and then normalizing the maximum value in each subplot to one. **e-j:** Fixation distance distribution to image center within the initial 11 fixations after the first saccade. **k:** Bhattacharyya coefficient (B_c) between models' and subjects' distributions of fixation distance to image center of the initial 20 fixations after the first saccade. Dots represent raw data; curves represent quadratic functions fitted to the dots.

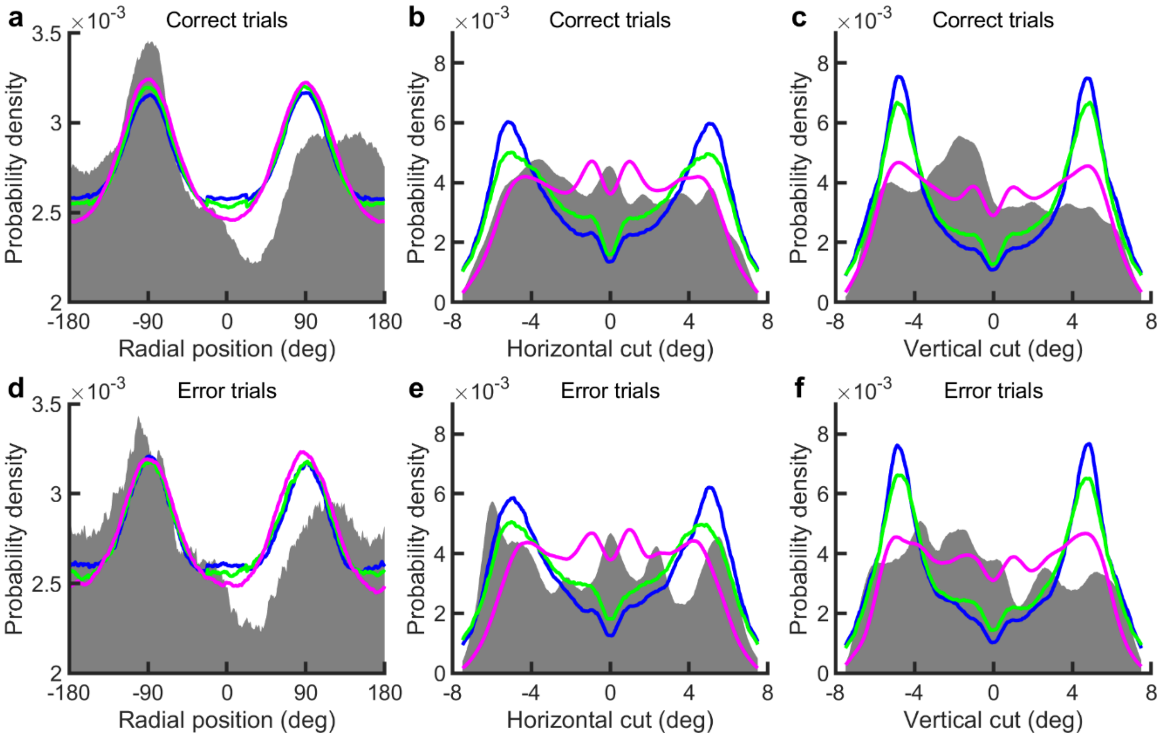

Supplementary Fig 8. **a**: Direction histograms of fixation location relative to the image center in correct trials. The histograms were obtained with a sliding radial window with a width of 45° centered different radial positions (x-axis). Rightward direction is 0° and upward direction is −90°. **b**: Horizontal cuts (through the center) through the fixation densities of correct trials (Fig 3a-d in the main text). Rightward direction is positive in x-axis. **c**: Vertical cuts (through the center) through the fixation densities of correct trials (Fig 3a-d in the main text). Upward direction is negative in x-axis. **d-f**: same as panels a-c but the analysis is done for data in error trials. In all panels area under curve are normalized to one.

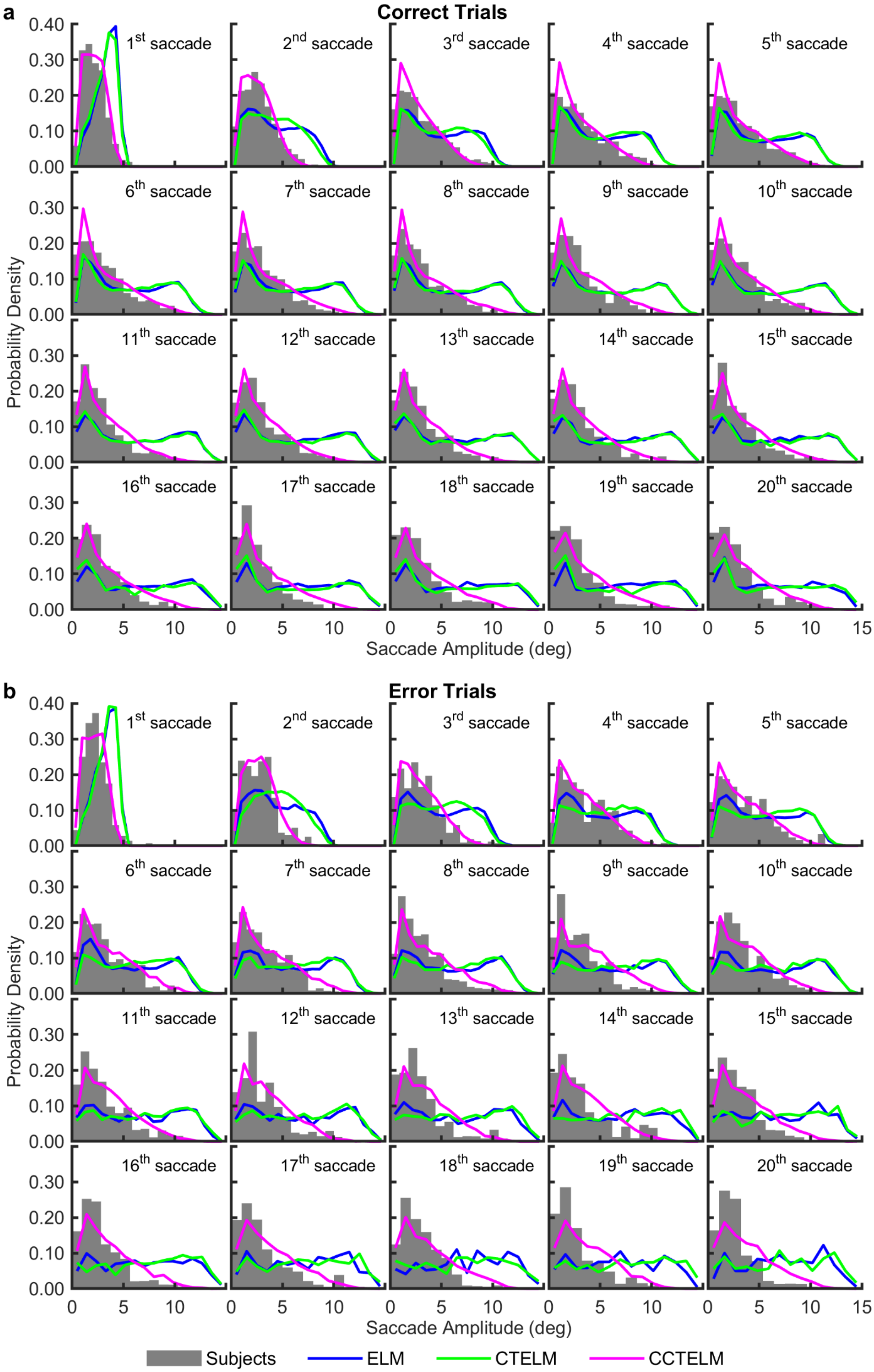
a
Correct Trials
1st saccade
2nd saccade
3rd saccade
4th saccade
5th saccade
6th saccade
7th saccade
8th saccade
9th saccade
10th saccade
11th saccade
12th saccade
13th saccade
14th saccade
15th saccade
16th saccade
17th saccade
18th saccade
19th saccade
20th saccade
Probability Density
Saccade Amplitude (deg)
b
Error Trials
Subjects
ELM
CTELM
CCTELM

Supplementary Fig 9. Distribution of saccade amplitude as a function of ordinal position in a sequence of saccades from all correct (**a**) and error (**b**) trials.

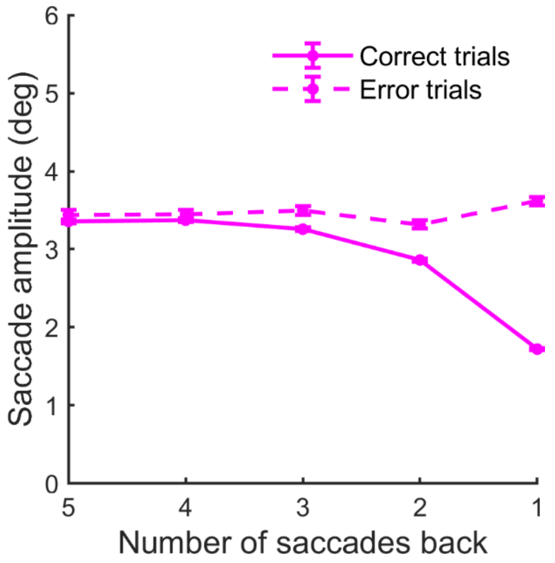

Supplementary Fig 10. Relationship between average saccade amplitude and the number of saccades back before the final response of the CCTELM model in correct and error trials. Error bar represents 99% confidence interval. The simulation results came from 100000 independently simulated trials.

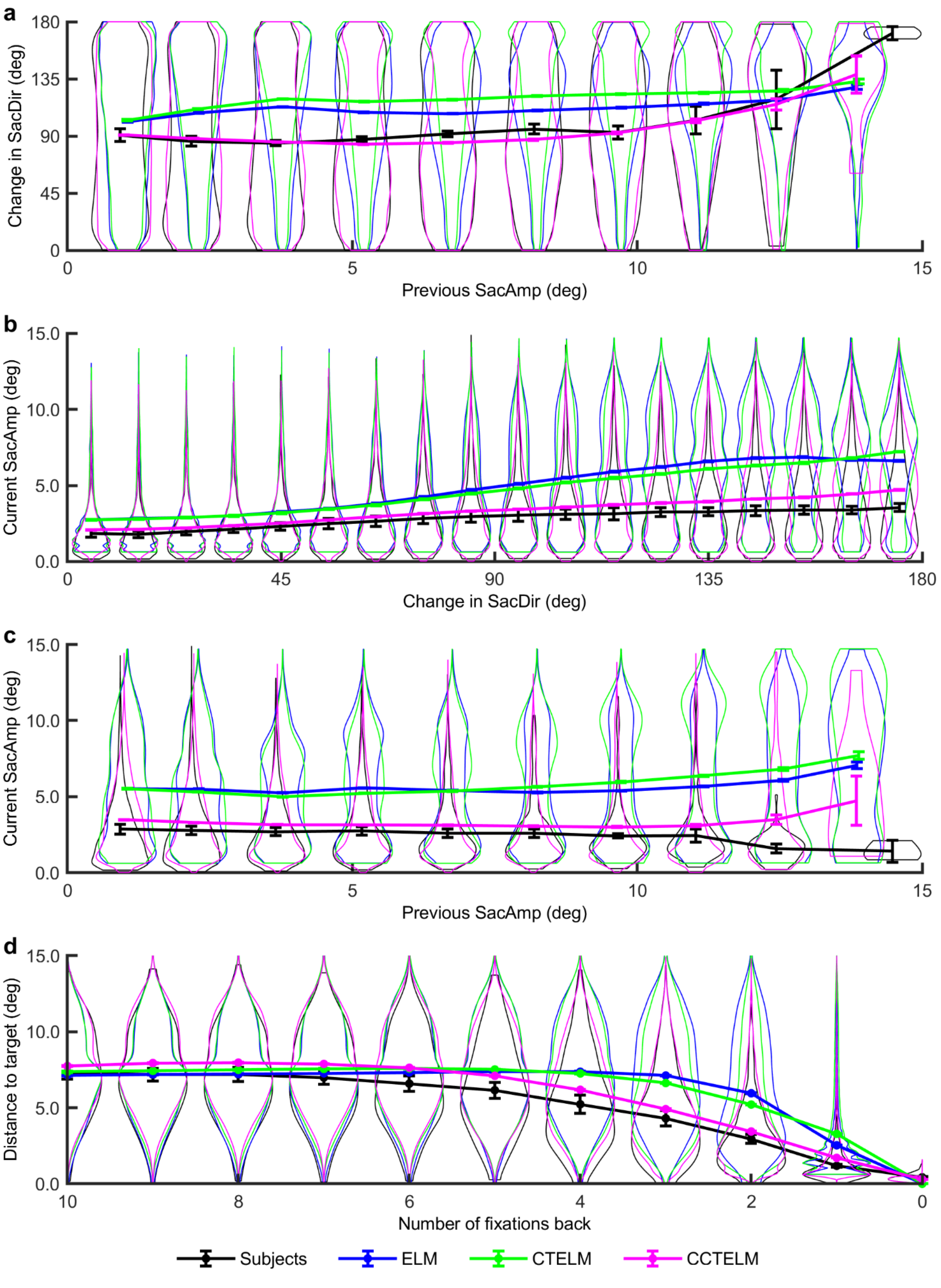

Supplementary Fig 11. Dependency between metrics of successive saccades and the speed of approaching the target within the final 10 fixations in correct trials. **a**: Relationship between the first saccade's amplitude (SacAmp) in two

consecutive saccades and the change in saccade direction (SacDir) in all trials. **b**: Relationship between the second saccade's amplitude in two consecutive saccades and the change in saccade direction in all trials. **c**: Relationship between the second and the first saccade's amplitude in two consecutive saccades in all trials. **d**: The distance between fixation location and the target location as a function of the number of fixations before correctly finding the target. Error bar represents 95% confidence interval. For experimental data $n = 6$ subjects. The simulation results came from 100000 independently simulated trials. The superimposed violin plots represent the distribution of the metrics in y axis grouped into each bin of the metrics in x axis.

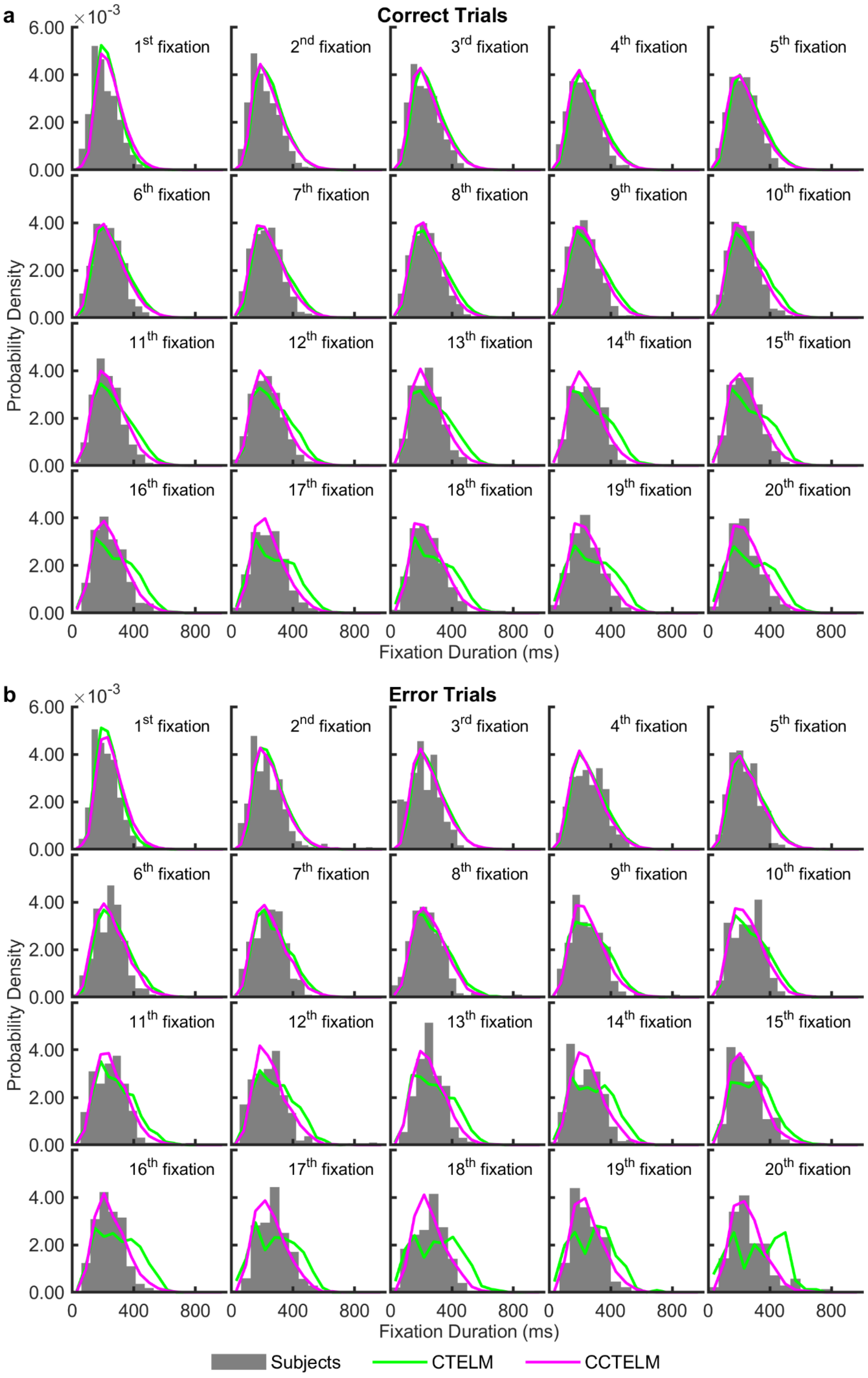
a
Correct Trials
×10-3
6.00
4.00
2.00
0.00
1st fixation
2nd fixation
3rd fixation
4th fixation
5th fixation
6th fixation
7th fixation
8th fixation
9th fixation
10th fixation
11th fixation
12th fixation
13th fixation
14th fixation
15th fixation
16th fixation
17th fixation
18th fixation
19th fixation
20th fixation
Probability Density
0
400
800
Fixation Duration (ms)
b
Error Trials
Subjects
CTELM
CCTELM

Supplementary Fig 12. Distribution of fixation duration as a function of ordinal position in a sequence of fixations from all correct (**a**) and error (**b**) trials.

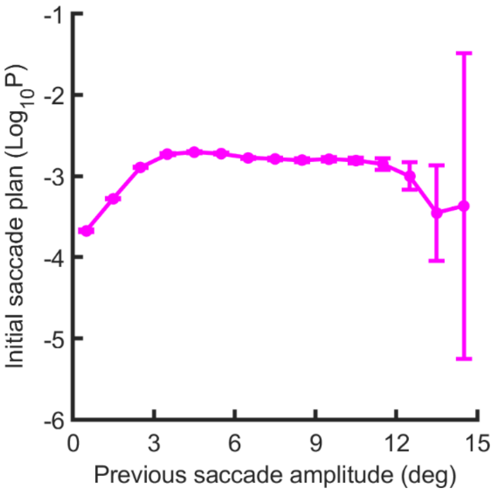

Supplementary Fig 13. Relationship between the change in saccade amplitude and the preview benefit of the next fixation in CCTELM model. Preview benefit is calculated as initial value of the next saccade's decision process (the posterior probability of target being at current attention location in logarithm scale). Lower initial value was closer to the saccade decision threshold and had larger preview benefit. Error bar shows 95% confidence interval. The simulation results came from 100000 independently simulated trials.

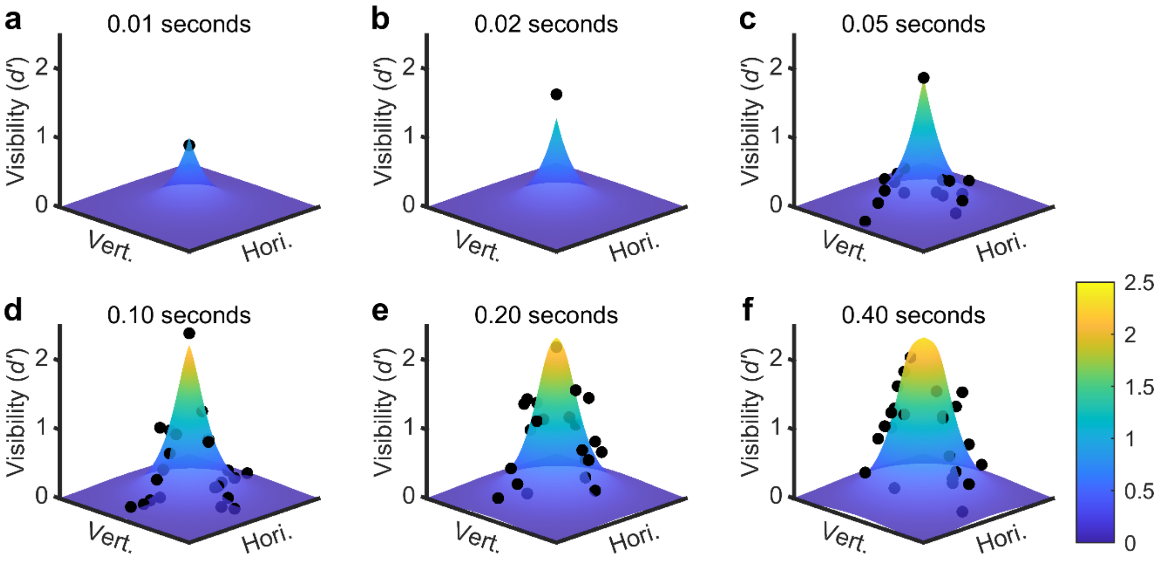

Supplementary Fig 14. The temporal dynamics of target visibility map without target location cue in the detection experiment. Data from all 4 subjects were combined according to measured location. Each subplot shows target visibility map after different stimulus exposure time in the detection task. Black dots are raw data pooled from the four subjects. The surface is the visibility map function (equations (10), (11), (12) in the main text) fit to the raw data. Not all locations were tested in these exposure time, so the number of black dots may differ in each subplot. Hori/Vert: horizontal/vertical dimension of the search field. Fixation location was at the center of the search field.

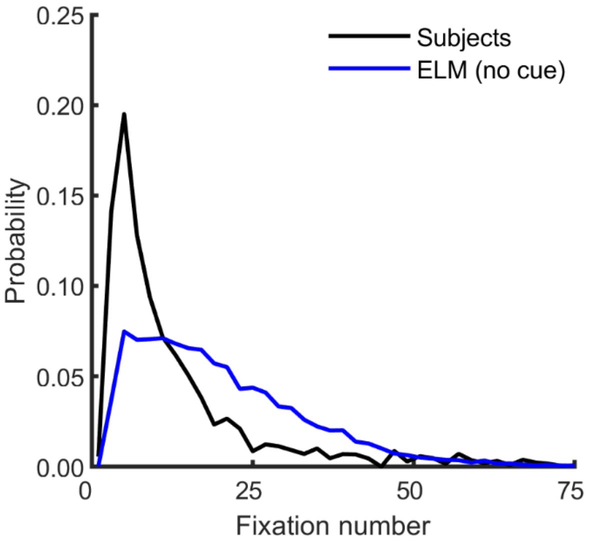

Supplementary Fig 15. The distribution of the number of fixations needed to find the target of subjects and the ELM model. The ELM model used the visibility map measured without target location cue (models in the main text used visibility map measured with target location cue). Subjects' data came from the 4 subjects that measured visibility map without target location cue (Supplementary Table 2). The model was simulated for 10000 trials and the correct response rate was 88.8%.

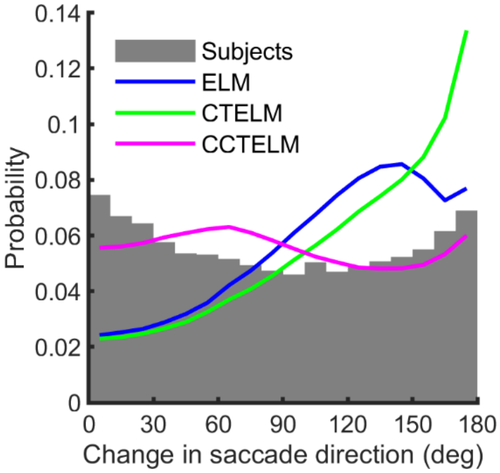

Supplementary Fig 16. Distribution of change in saccade direction between two consecutive saccades of humans and the three models in all trials.

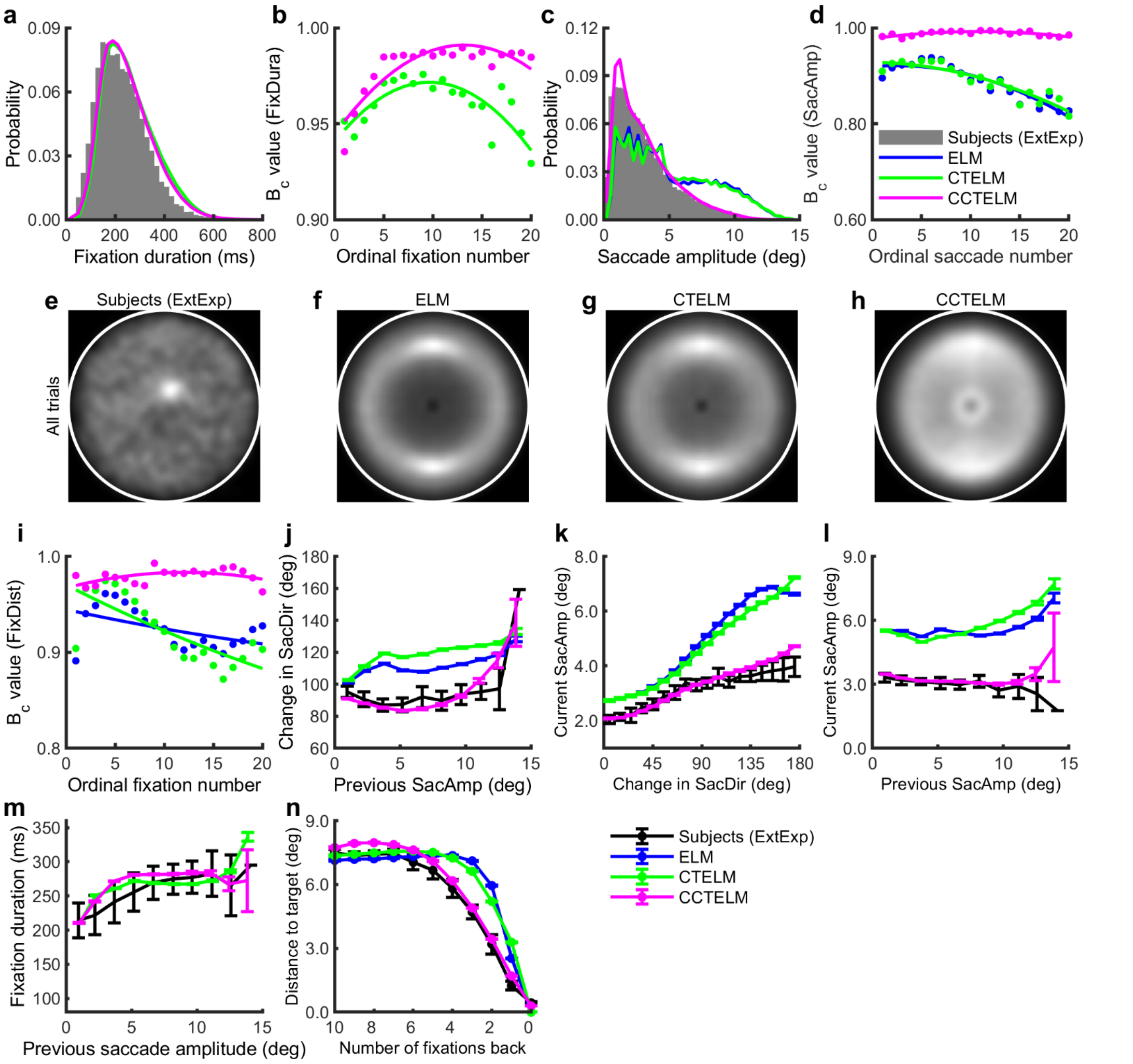

Supplementary Fig 17. Comparison of the CCTELM model (with the same parameter values) to data from an extended visual search experiment (ExtExp) with a higher target RMS contrast (0.15). Both correct and error trials were included in the analysis. Each eye movement metrics was first summarized within each subject's data and then average across subjects. **a**. The distribution of fixation duration. **b**. Bhattacharyya coefficient (B_c) between the models' and subjects' fixation duration distributions of the initial 20 fixations after the first saccade. **c**. The distribution of saccade amplitude. **d**. B_c between the models' and subjects' saccade amplitude distribution of the initial 20 saccades. **e-h**. Distribution of fixation location in the search field (inside the white circle), lighter means higher density. **i**. B_c between models' and

subjects' distributions of fixation distance to image center of the initial 20 fixations after the first saccade. **j**: Relationship between the first saccade's amplitude (SacAmp) in two consecutive saccades and the change in saccade direction (SacDir). **k**: Relationship between the second saccade's amplitude in two consecutive saccades and the change in saccade direction. **l**: Relationship between the second and the first saccade's amplitude in two consecutive saccades. **m**. Relationship between fixation duration and previous saccade amplitude. **n**. The distance between fixation location and the target location as a function of the number of fixations before correctly finding the target. Error bar represents 95% confidence interval. For experimental data $n = 7$ subjects. The simulation results came from 100000 independently simulated trials.

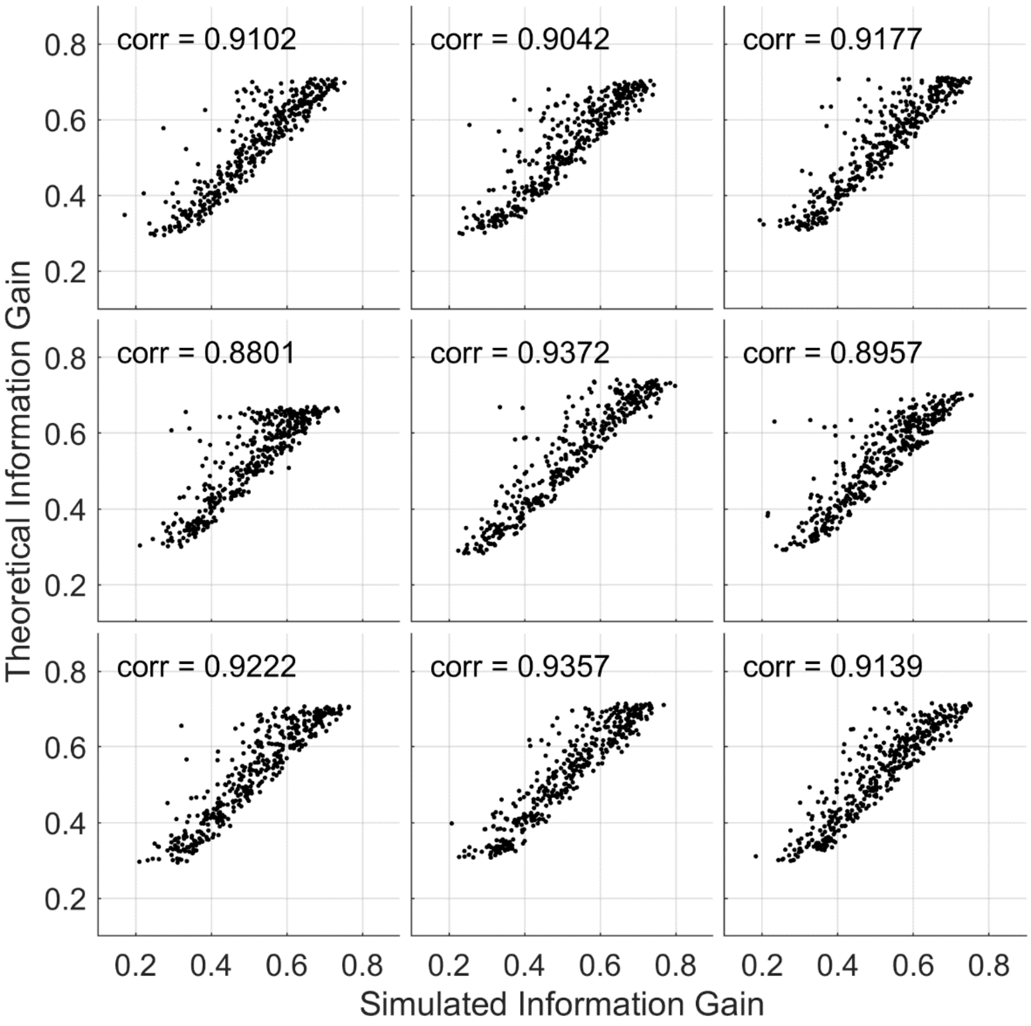

Supplementary Fig 18. The correlation between expected (vertical axis) and actual (horizontal axis) information gain from the results of randomly selected 9 simulation sessions. Each dot in a plot represents one possible fixation locations, so there are 400 dots in each plot. The Pearson correlation coefficients are shown at the upper left of each plot.

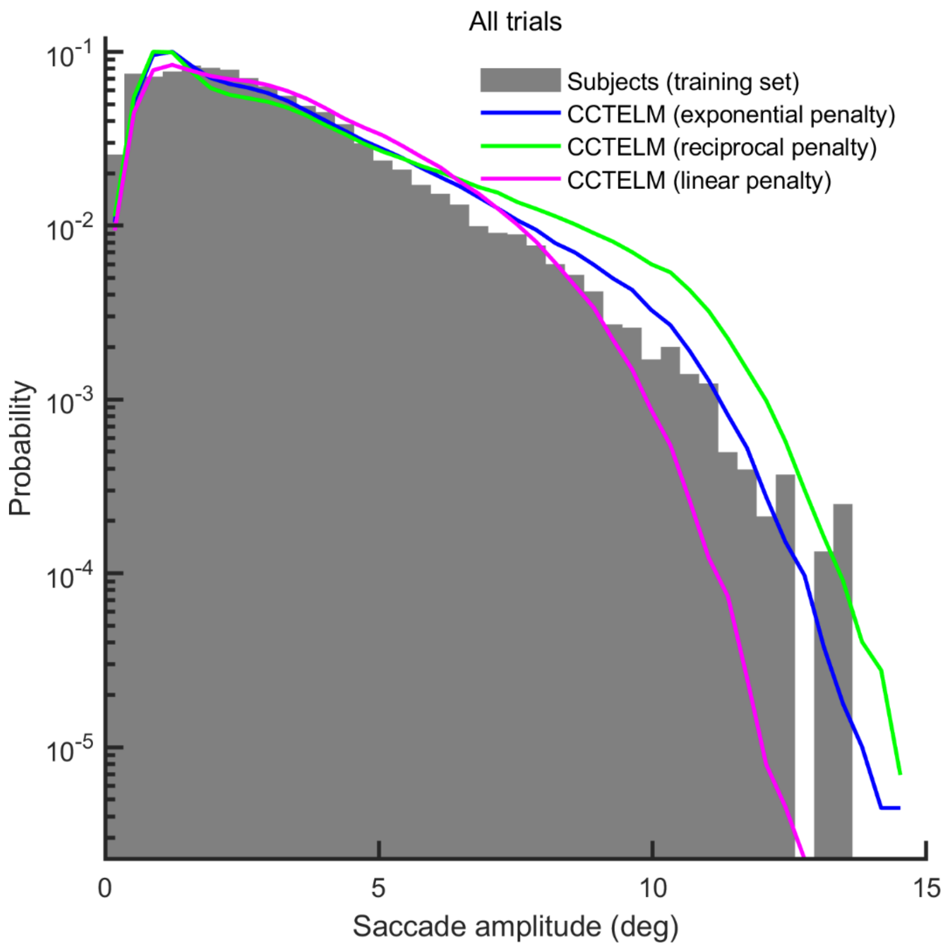

Supplementary Fig 19. Comparison of three forms of saccade amplitude penalty function. The exponential penalty is equation (26) in the main text. The reciprocal penalty function is $H(x) = c/(\max(x,1)+c)$ where x is the distance between current fixation location and potential next fixation location, and c is the parameter. The linear penalty function is $H(x) = \max(1-\max(x,1)/c)$. The parameters in these three CCTELM models (each with a different penalty function) were fit by genetic algorithm and evaluated for 100000 trials and compared against the distribution of saccade amplitude of subjects in training set.

Parameters used in the three visual search models

Model	Parameters	Description	Value	Determination
Shared	p_1, p_2, p_3, p_4, p_5	Parameters in visibility map function.	19.683, 0.00889, 43.195, 0.0153, 1.635	Fit by GlobalSearch algorithm
	n	Number of potential target locations.	400	A priori
	dt*	Time step in seconds.	0.001	A priori
	Normal saccade frequency *	The probability that a saccade is triggered by the main decision process.	97%	A priori, according to low-latency saccade frequency
	Low-latency saccade frequency *	The probability that a saccade is not triggered the main decision process.	3%	A priori [3]
	Latency distribution of low-latency saccades (seconds) *	The latency from previous saccade decision to the time when eye starts to move in the following saccade.	$\Gamma\left(10, 6.9\times10^{-3}\right)$ $+0.03$	A priori [4]
	Eye-brain lag (seconds) *	Time delay from retina to primary visual cortex.	0.06	A priori [5]
	Saccade lag (seconds) *	Time delay from frontal eye field to eye movement muscle.	0.03	A priori [6]
ELM	θ_T	Target detection threshold.	0.240	A priori (Fit by human correct response rate)
CTELM	q_1, q_2, q_3	Parameters in saccade threshold function.	-9.564, 0.508, 1.063	Fit by genetic algorithm
	θ_T	Target detection threshold.	0.982	A priori (Fit by human correct response rate)
CCTELM	q_1, q_2, q_3	Parameters in saccade threshold function.	-17.815, 0.199, 0.498	Fit by genetic algorithm
	c	Parameters in saccade amplitude penalty function.	433.09	Fit by genetic algorithm
	θ_T	Target detection threshold.	0.952	A priori (Fit by human correct response rate)
	M	Number of previous fixations that the model can keep in memory.	8	A priori (Fit by sequential eye movement metrics)

Supplementary Table 1. Parameters used in the three visual search models. The value of a priori parameters were determined prior to fitting of other parameters by genetic algorithm. Shared parameters were used in all models except specifically indicated ones. *: Not used in the ELM model.

Individual characteristics and experiment information of each subject

Index	Gender	Age	Target RMS contrast	Detection task version	Visibility map trial number	Visual search trial number	Visual search fixation number	Visual search saccade number	Visual search percent correct	Parameter fitting
1	Male	23	0.11135	Cued, Not cued	8850	400	7046	6567	87.25%	Training
2	Female	23	0.12028	Cued	5500	260	3012	2612	89.23%	Training
3	Female	22	0.12517	Cued	5100	330	8120	7461	87.88%	Training
4	Male	26	0.12537	Cued	5400	200	3261	3005	88.00%	Training
5	Male	24	0.11506	Fast	1500	300	9234	8870	76.67%	Testing
6	Female	30	0.12589	Fast	1500	500	10101	8952	80.40%	Testing
7	Male	25	0.12661	Fast	1500	198	7464	6990	81.31%	Testing
8	Female	23	0.12870	Not cued	4850	200	5617	4724	76.00%	Testing
9	Male	22	0.13520	Not cued	5300	260	2873	2581	95.77%	Testing
10	Male	19	0.13545	Not cued	5350	500	9031	8073	93.00%	Testing

Supplementary Table 2. Individual characteristics and experiment information of each subject. In detection task version, “cued” is the first version mentioned in method section, “not cued” is the second version, and “fast” is the third version. The visibility map trial number includes the trials to select target RMS contrast and trials in the detection task. The fixation number in visual search includes the first fixation at the image center and last response fixations of each trial. Some of these fixations were discarded when summarizing certain fixation metrics (see methods). The parameter fitting column indicates whether the subject’s eye movement data was used to train or test the visual search model.

Supplementary Reference

1. Nuthmann A, Vitu F, Engbert R, Kliegl R. No evidence for a saccadic range effect for visually guided and memory-guided Saccades in simple saccade-targeting tasks. PLoS One. 2016;11(9):e0162449.
2. Najemnik J, Geisler WS. Simple summation rule for optimal fixation selection in visual search. Vision Res. 2009;49(10):1286–94.
3. Munoz DP, Broughton JR, Goldring JE, Armstrong IT. Age-related performance of human subjects on saccadic eye movement tasks. Exp Brain Res. 1998;121(4):391–400.
4. Fischer B, Ramsperger E. Human express saccades: effects of randomization and daily practice. Exp Brain Res. 1986;64(3):569–78.
5. Nowak LG, Bullier J. The Timing of Information Transfer in the Visual System. In: Rockland KS, Kaas JH, Peters A, editors. Extrastriate Cortex in Primates [Internet]. Boston, MA: Springer US; 1997. p. 205–41. Available from: https://doi.org/10.1007/978-1-4757-9625-4_5
6. Bruce CJ, Goldberg ME, Bushnell MC, Stanton GB. Primate frontal eye fields. II. Physiological and anatomical correlates of electrically evoked eye movements. J Neurophysiol [Internet]. 2017;54(3):714–34. Available from: https://doi.org/10.1152/jn.1985.54.3.714
